# QM/MM Molecular Dynamics Simulations Revealed Catalytic Mechanism of Urease

**DOI:** 10.1021/acs.jpcb.1c10200

**Published:** 2022-03-03

**Authors:** Toru Saito, Yu Takano

**Affiliations:** Department of Biomedical Information Sciences, Graduate School of Information Sciences, Hiroshima City University, 3-4-1 Ozuka-Higashi, Asa-Minami-Ku, Hiroshima 731-3194 Japan

## Abstract

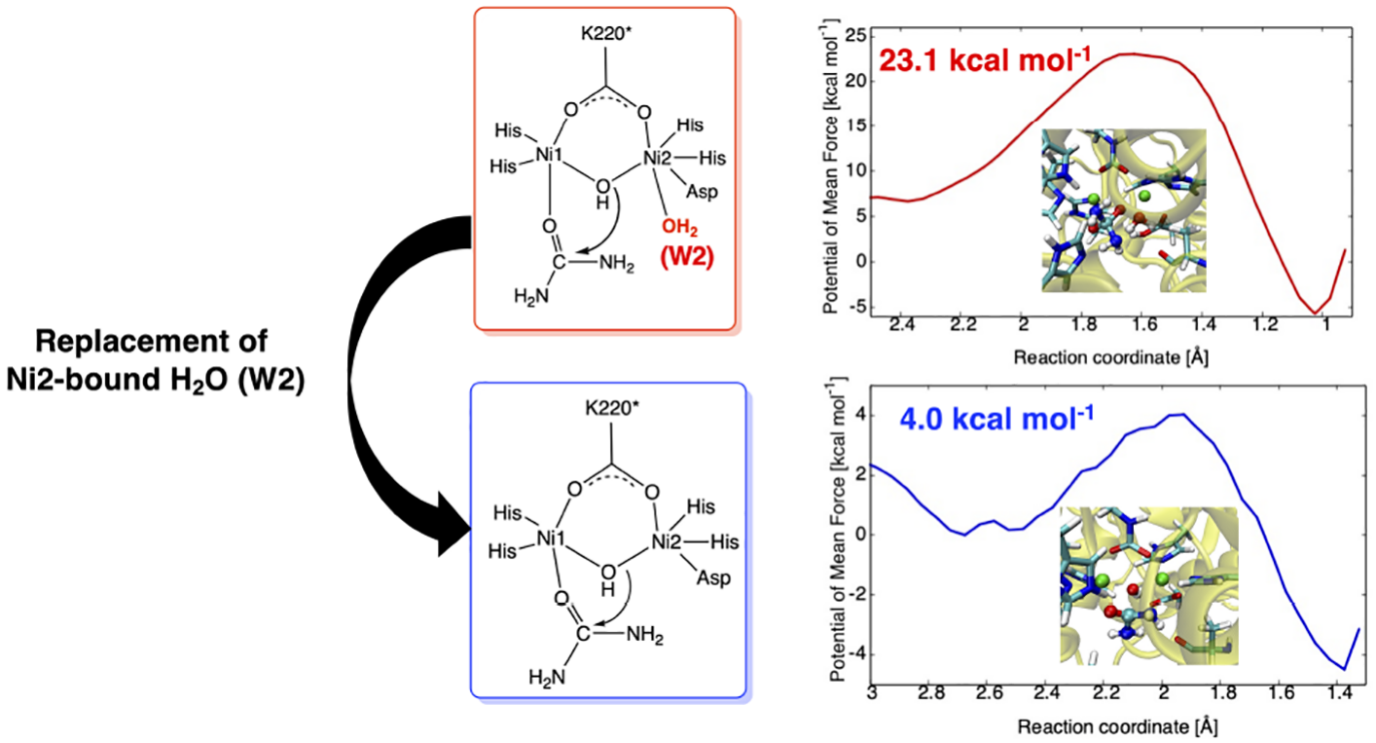

Urease catalyzes the hydrolysis of urea to form ammonia and carbamate, inducing an overall pH increase that affects both human health and agriculture. Inhibition, mutagenesis, and kinetic studies have provided insights into its enzymatic role, but there have been debates on the substrate binding mode as well as the reaction mechanism. In the present study, we report quatum mechanics-only (QM-only) and quantum mechanics/molecular mechanics molecular dynamics (QM/MM MD) calculations on urease that mainly investigate the binding mode of urea and the mechanism of the urease-catalyzed hydrolysis reaction. Comparison between the experimental data and our QM(GFN2-xTB)/MM metadynamics results demonstrates that urea hydrolysis via a complex with bidentate-bound urea is much more favorable than via that with monodentate-bound urea for both nucleophilic attack and the subsequent proton transfer steps. We also indicate that the bidentate coordination of urea fits the active site with a closed conformation of the mobile flap and can facilitate the stabilization of transition states and intermediates by forming multiple hydrogen bonds with certain active site residues.

## 1 Introduction

**Scheme 1:**
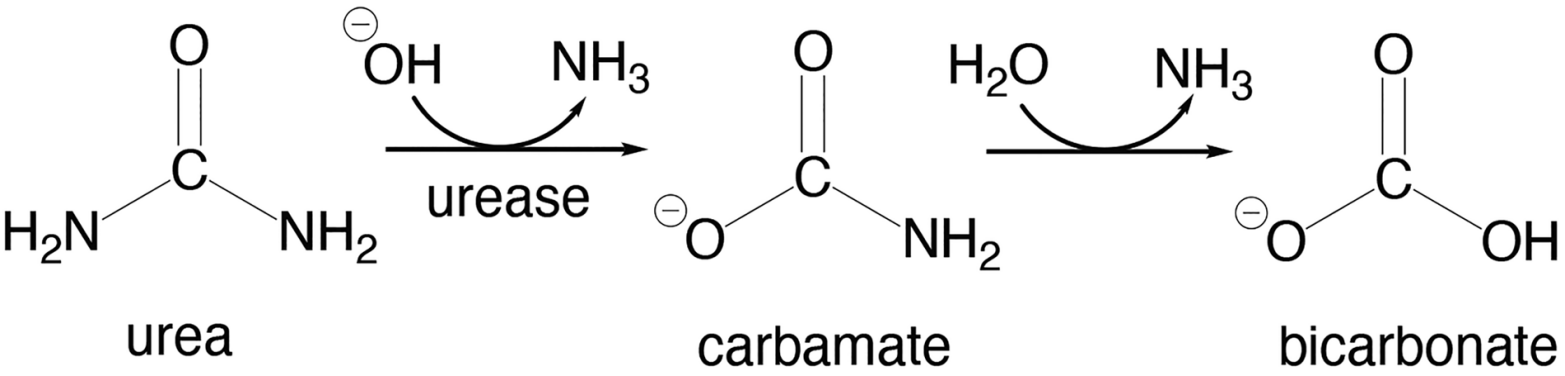
Urea Hydrolysis Reaction

Urease, a nickel-containing metalloenzyme, is found in various organisms such as plants, algae, fungi, and prokaryotes.^1−4^ The binuclear Ni center in its active site catalyzes urea hydrolysis to form ammonia (NH_3_) and carbamate, which spontaneously decomposes into another NH_3_ molecule and bicarbonate (Scheme 1).^5−9^ This enzyme exhibits a rate enhancement of ca. 10^14^-fold over the uncatalyzed reaction.

The enzymatic hydrolysis of urea leads to an overall pH increase that affects both human health and agriculture.^10−13^ The human pathogenic bacterium *Helicobacter pylori* is classified as the most important risk factor for gastric cancer. The urease produced by *H. pylori* plays an essential role for the colonization of the stomach by neutralizing the acidic environment. Concerning urease reactions in soils, the NH_3_ volatilization causes the loss of efficacy of urea fertilizer applications.

**Figure 1:**
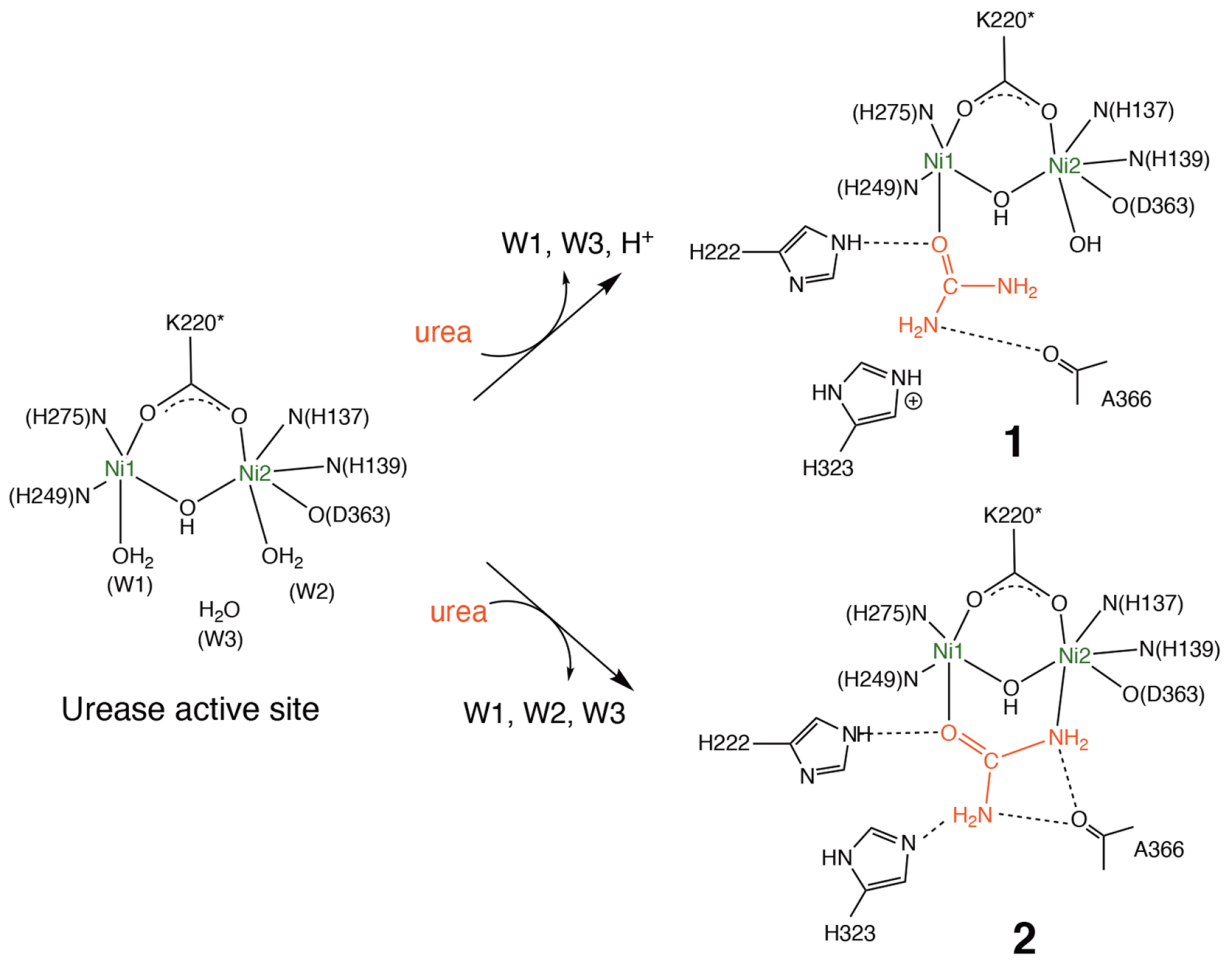
Active site of *Sporosarcina pasteurii* urease (SPU) and two possible substrate binding modes, monodentate **1** and bidentate **2**.

To design effective inhibitors of urease, numerous crystal structures have been determined both in the absence and in the presence of inhibitors.^14−24^ It has been revealed that two divalent nickel ions in the active site, namely, Ni1 and Ni2, are separated by ca. 3.5 Å and coordinated to two bridging ligands, a hydroxide and a carbamylated lysine denoted as K220*, as illustrated in Figure 1. Two histidine residues (H249 and H275) and a water molecule (W1) are coordinated to Ni1, while two histidines (H137 and H139), a monodentate aspartate acid residue (D363), and a water molecule (W2) are bound to Ni2. These water molecules interact with a nearby water molecule (W3) via a strong hydrogen bond. A consensus regarding the enzymatic reaction has been reached that W1 and W3 are initially displaced and the Lewis acid character of the coordinatively unsaturated Ni1 site enhances the electrophilicity of the carbonyl O atom of urea, leading to the formation of the Ni1–O(urea) bond. However, information from the inhibitor bound crystal structures is not sufficient to assess whether urea binds to the active center in such a monodentate manner or a bidentate complex is formed by further replacement of W2 (Figure 1). Thus, several reaction pathways have been proposed for the subsequent catalytic reaction steps.^9,15,25−27^

**Scheme 2:**
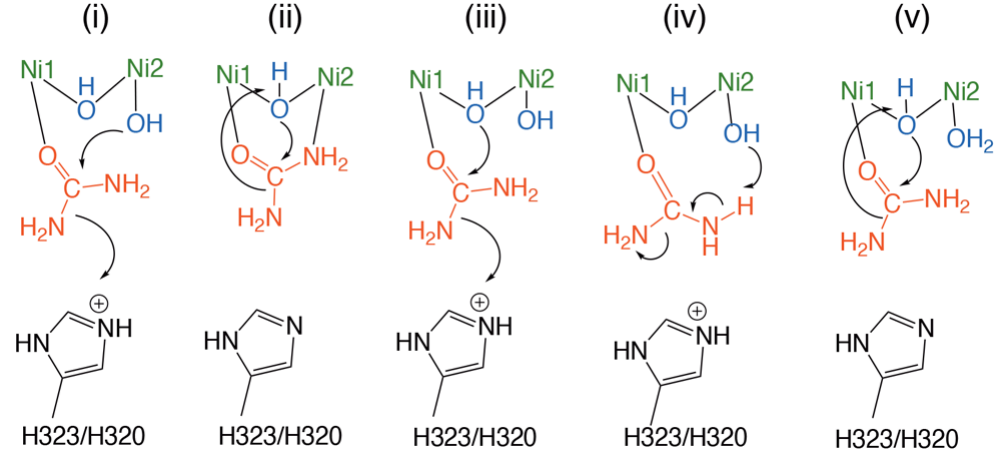
Previously Proposed Urease Reaction Mechanisms (i)–(iv) and Our Proposed One (v), the Details of Which Are Described in the Text, with Amino Acid Numbering for SPU/KAU

Scheme 2 summarizes possible reaction mechanisms (i)–(iv) proposed so far. Inspired by the crystal structures of native *Klebsiella aerogenes* (KAU), mutagenesis, and kinetic studies, Hausinger and co-workers suggested that urea binds to the active site in a monodentate manner, referred to herein as mechanism (i).^14,25^ In mechanism (i), Ni2 enhances the nucleophilicity of W2 by deprotonation, and the doubly protonated H320 (KAU numbering) acts as a general acid. The reaction can proceed through nucleophilic attack of the formed terminal hydroxide ion on the carbonyl C atom of urea to give a tetrahedral intermediate, followed by a proton transfer from H320 to the urea NH_2_ group. Ciurli and co-workers alternatively proposed mechanism (ii) starting from a bidentate complex on the basis of the crystal structures of native and inhibitor-bound *Sporosarcina pasteurii* urease (SPU).^15,19,20,23^ The recently determined urea bound SPU complex inhibited by fluoride supports mechanism (ii),^24^ in which the bridging hydroxide ion acts as both a nucleophile and a general acid.

It should be noted that both the steric structures and the active site environments of SPU and KAU are similar, though the protonation state of H323/H320 (SPU/KAU numbering) and positions of nearby residues are different due to the different pH conditions for crystallization. A mixture of mechanisms (i) and (ii) has also proposed as mechanism (iii), in which the bridging hydroxide ion is a nucleophile and H323/H320 acts as a general acid.^26^

In analogy with other metalloenzymes containing only nickel,^28−31^ a few computational investigations have also been reported with active site cluster models.^32−34^ Suárez et al.^32^ have performed density functional theory (DFT) calculations on small active site models to determine the most likely reaction mechanism. They have compared a variant of mechanism (i), where the terminal OH is replaced by H_2_O with mechanism (ii), and concluded that mechanism (ii) is favorable on the basis of computed barrier heights. Apart from mechanisms involving nucleophilic attack, Estiu and Merz have proposed an elimination mechanism (iv)^33^ as with a biomimetic study,^35^ but a cyanate intermediate has never been experimentally identified in the catalytic process. In this way, these previous computational studies did not attempt to compare four mechanisms (i)–(iv) explicitly. One must also keep in mind that it is essential that the effect of protein environment on the catalytic activity is taken into account explicitly, not to mention active site residues such as H323/H320.

Here, quantum mechanics/molecular mechanics molecular dynamics (QM/MM MD) simulations^36,37^ were applied to avoid a pitfall of interpretation arising from the use of small cluster models. The present study focuses on the binding mode of urea to the dinickel center and reinvestigation of the urease-catalyzed reaction by comparing four mechanisms (i)–(iv). We wish to settle a long-standing controversy, providing mechanistic insights into the urease-catalyzed reaction.

## 2 Computational Method

### 2.1 System Setup

To prepare monodentate (**1**) and bidentate (**2**) complexes shown in Figure 1, the X-ray crystal structure of the SPU-urea complex refined at a resolution of 1.42 Å was taken from the Protein Data Bank (PDB code: 6QDY).^24^ We replaced the bridging fluoride in 6QDY with hydroxide ion and constructed an active site model for **2** consisting of urea, Ni1, Ni2, a bridging hydroxide ion, and side chains of residues coordinated to two Ni^2+^ ions (Figure S1). A monodentate counterpart with a terminal hydroxide ion (**1**) was also constructed to obtain appropriate initial positions of urea and the terminal hydroxide ion. All QM calculations were performed with the ORCA 4.2.1 program package.^38^ The geometries of the two active site models were fully optimized in the open-shell singlet state using the Gaussian version of UB3LYP method by using the UKS B3LYP/G keyword, together with the RIJCOSX approximation, the def2-SVP basis set, and the auxiliary def2/J basis set.^39−44^ Then, their atomic charges were obtained with the ChelpG procedure^45^ on the optimized structures (Figure S1). The CHARMM force field parameters for urea and K220* were generated by the CHARMM General Force Field program.^46^ The Lennard-Jones parameters proposed by Merz et al. was assigned to Ni^2+^ ions.^47^ The created topology and parameter files were presented in the Supporting Information. Hydrogen atoms were added by the CHARMM-GUI input generator,^48^ provided that the epsilon position of H222 was protonated for both **1** and **2**, and H323 was doubly protonated for **1**. The two systems were in turn solvated within a 100 × 110 × 100 Å rectangular box of water molecules and neutralized by Na^+^ ions using the Visual Molecular Dynamics (VMD) program,^49^ respectively.

### 2.2 Classical MD and QM/MM MD Equilibration

For **1** and **2**, each QM region contained the aforementioned active site complex, combined with A366 and side chains of H222 and H323, while the MM region was defined as the rest of the system as illustrated in Figure 1. Note that A366 and H222 were assumed to form hydrogen-bonding interactions with urea, stabilizing substrate binding and transition states.^9,50^ Classical 10 ns MD simulations were performed in the *NPT* ensemble at 300 K with a time step of 2.0 fs, using the NAMD program.^51^ Nonbonded interactions were cutoff at 14 Å with a switching function, and electrostatic interactions were evaluated by the particle mesh Ewald method.^52^ During the MD simulations, all the atoms in the QM region represented by the CHARMM General Force Field were virtually kept fixed with the constraintScaling of 50.0. The CHARMM36 force field and TIP3P water models were utilized to describe the MM region.^53^ The computed trajectories were listed in Figure S2. The QM/MM MD simulations were performed with NAMD. The tight-binding GFN2-xTB method^54^ was employed for the QM region using the ORCA 4.2.1 program package, while the MM subsystem was treated by the same force field parameters as the classical MD simulations. The NAMD-ORCA interface^55^ was exploited to compute the forces on the MM atoms. The QM-MM covalent boundary was treated by the link atom method. The total charge and spin multiplicity were set to 1 and 5 for **1** and 0 and 5 for **2** (see below). The two systems were equilibrated without any constraints for 2.0 ps.

### 2.3 QM-Only Cluster Calculations

Although broken-symmetry DFT calculations with localized basis sets may be useful for investigating urea hydrolysis catalyzed by the dinickel complex with an open-shell singlet ground state, the free energy analysis is still costly for the QM region sizes considered in this work (Figure 1). Alternatively, we chose to use GFN2-xTB, which is fast and can reasonably be accurate for transition metal complexes.^56^

We began by assessing whether the method can be alternative to broken-symmetry DFT calculations. The accuracy of GFN2-xTB for geometry optimization and the energetics of urea hydrolysis was validated by means of smaller cluster models for **1** and **2**. For comparison purposes, we used the UB3LYP/def2-SVP method as the reference method on the basis of previous studies of the structural and magnetic properties of the urease active site.^32,34,57,58^ An active site model without urea (**3**) was also tested in the case of a coordinatively unsaturated metal center. To minimize artificial intramolecular interactions, we constructed three QM-only cluster models **1**′-**3**′ in which the side chains of K220* and histidine residues were truncated as methylcarbamic acid and imidazole, respectively (Figure S3). The geometries for **1**′–**3**′ were fully optimized in the open-shell singlet state at the UB3LYP level. In the case of GFN2-xTB, both the quintet and closed-shell singlet states were tested for the optimizations because it gave only closed-shell configurations for the singlet. Then, the potential energy profiles of possible mechanisms starting with **1**′ and **2**′ were predicted by GFN2-xTB and UB3LYP/def2-SVP. All GFN2-xTB calculations were performed with the ORCA 4.2.1.^38^ The Gaussian16 program package^59^ was applied to the UB3LYP/def2-SVP method for the purpose of locating saddle points efficiently. Note that we verified that the UB3LYP/def2-SVP implemented in Gaussian16 provided comparable results to that in ORCA 4.2.1.

### 2.4 GFN2-xTB/MM Metadynamics Simulations

Subsequently, the QM(GFN2-xTB)/MM(CHARMM36) metadynamics method^60^ was performed by the collective variables module of NAMD with the NAMD-ORCA interface.^55^ One-dimensional potentials of mean force (1D-PMFs) for the enzymatic processes were calculated in the *NVT* ensemble at 300 K. The C(urea)–O(hydroxide) bond distance, in a range from 1.30 to 3.40 Å, was chosen as the reaction coordinate for nucleophilic attack to form a tetrahedral intermediate. When a proton transfer driven by hydroxide ion was calculated, the reaction coordinate was set to the H(hydroxide)–N(urea) separation ranging from 0.90 to 2.60 Å. For all cases, a hill weight of 0.30 kcal/mol, a hill width of 2.0 Å, and a hill frequency of 50 fs was used. The simulations were performed for 20–30 ps with a 0.5 fs time step of integration.

## 3 Results and Discussion

### 3.1 Possible Mechanisms Starting with 1 and 2

**Figure 2:**
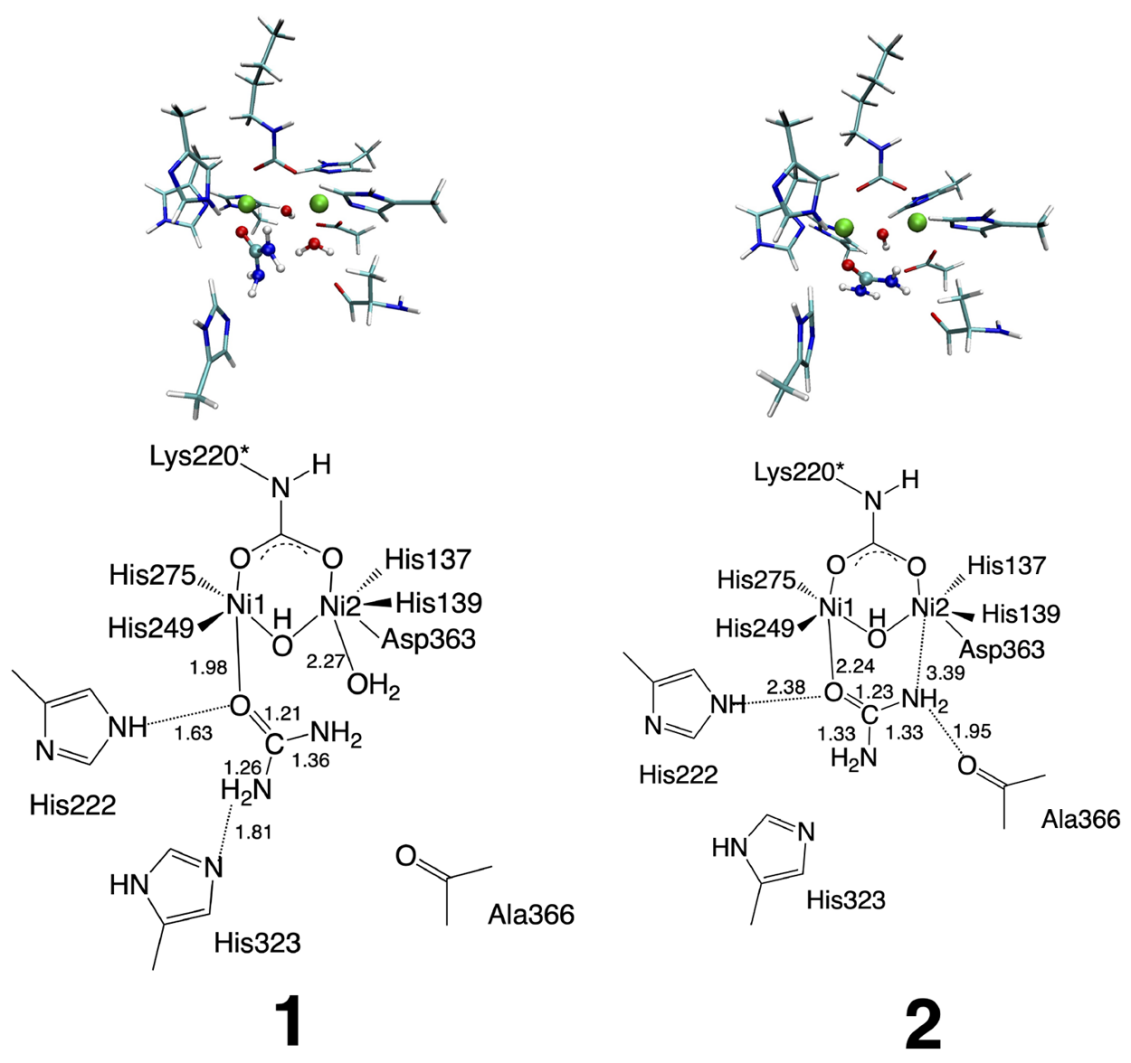
QM regions of **1** and **2** after the QM(GFN2-xTB)/MM MD equilibrations (top). Key bond distances are also presented in Å (bottom).

The orientation of urea in **1** before the QM(GFN2-xTB)/MM MD equilibration was suitable for nucleophilic attack of both the terminal and bridging hydroxide ions, with the O···C(urea) separations of 2.37 and 3.40 Å, respectively. The position of H323 was also close to urea consistent with a closed conformation found in KAU, and it would act as a general acid capable of producing leaving ammonia.^14,25^ However, the QM(GFN2-xTB)/MM MD equilibration led to a significant structural change. A proton in the proximal NH_2_ group of urea was spontaneously transferred to the Ni2-bound hydroxide ion, followed by the proton transfer from the doubly protonated H323 to the same amide nitrogen atom. Consequently, the active site complex resulted in having a terminal water ligand W2 and H323 in the neutral form as shown in Figure 2.

These trends substantially differed from those in a previous QM study using small active site models^33^ in that the activated terminal hydroxide ion spontaneously returned to W2 and thus had no possibility to attack the carbon atom of urea.

The significant structural change found in **1** motivated us to perform an additional calculation using a more accurate broken-symmetry DFT method to judge whether the GFN2-xTB/MM-based equilibration is efficient and reliable. Specifically, the geometry of the QM region in the open-shell singlet state was optimized at the UB3LYP/def2-SV(P) coupled with the RIJCOSX approximation and the def2/J auxiliary basis set. The UB3LYP optimized structure also turned out to be the same as the result of the GFN2-xTB/MM MD equilibration (Figure S4), which reassured us that the activated hydroxide ion coordinated to Ni2 in **1** could act as a base and not a nucleophile regardless of the chosen QM methods. It was also shown that mechanism (iv) was not likely to occur in the presence of protein environment including H323, in agreement with the fact that a cyanate intermediate had never been observed experimentally. By contrast, structural changes such as a bond scission/formation were not observed in **2** during the equilibration (Figure 2).

In this context, urea hydrolysis could proceed through the following two reaction pathways. The first one is the nucleophilic attack of the bridging hydroxide ion on the carbon atom of urea in **1**, while W2 acted as a spectator, referred to herein as mechanism (v) (Scheme 2). An analogous mechanism was recently reported by Netto and co-workers.^61^ The second one is mechanism (ii) starting with **2**.

### 3.2 Potential Energy Profiles Obtained from QM-Only Cluster Calculations

The differences between the closed-shell singlet and quintet states in the optimized geometries of **1**′ and **2**′ are comparable. The computed RMSD values for the singlet (quintet) state geometries with respect to the UB3LYP-optimized structures are 0.72 (0.76) and 0.74 (0.68) Å for **1**′ and **2**′, respectively. In the case of **3**′, the RMSD value of 0.28 Å for the quintet state geometry is much smaller than that of 1.08 Å for the singlet state geometry (see also Figure S3). We suggest that the GFN2-xTB calculations in the quintet state are superior to those in the closed-shell singlet state, and thus the quintet-state potential energy surfaces of possible reactions are presented hereafter.

**Figure 3:**
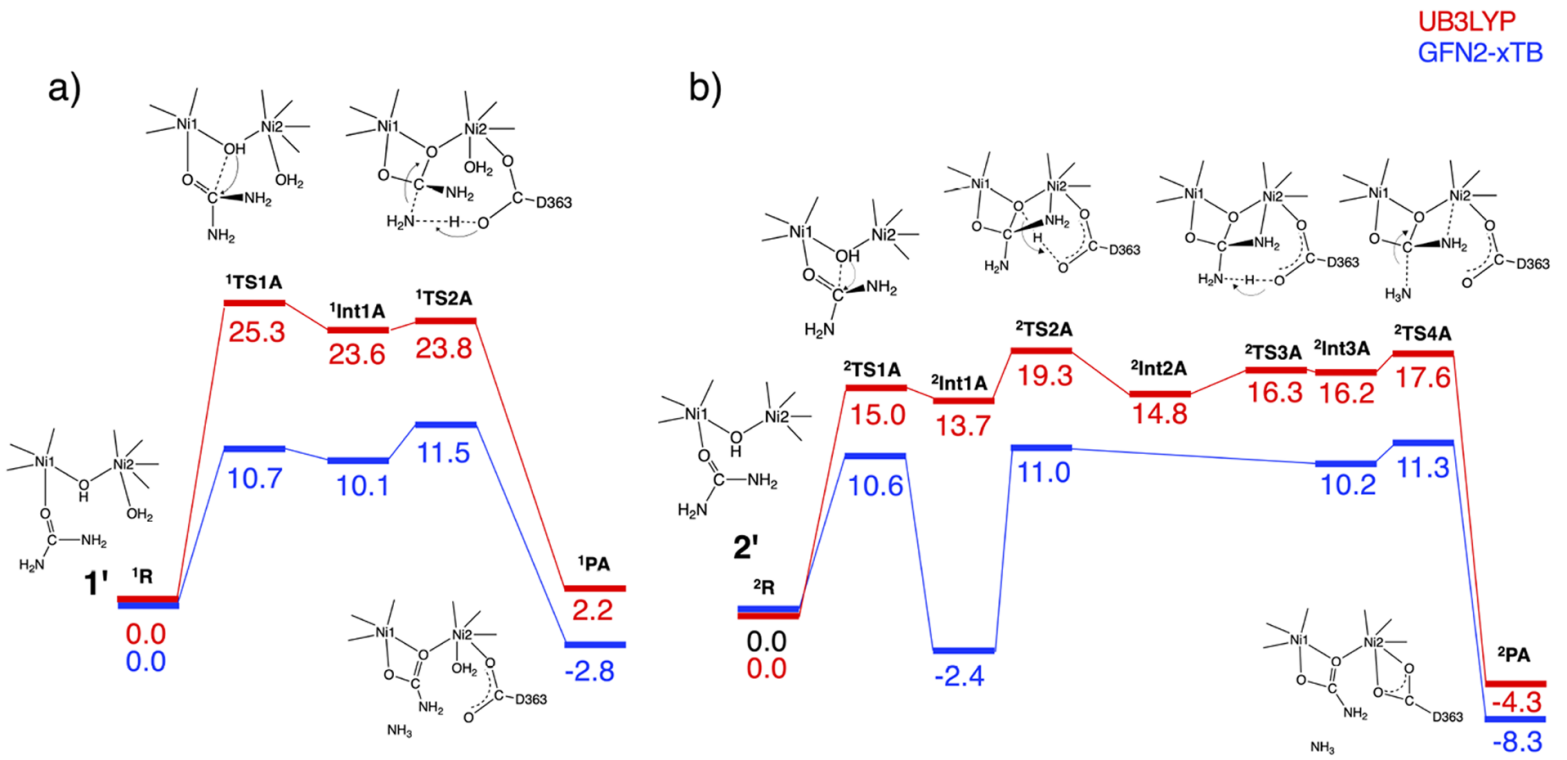
Potential energy profiles (in kcal mol^–1^) and illustrations of the transition state and product structures corresponding to (a) mechanism (v) starting from **1**′ and (b) mechanism (ii) starting from **2**′ obtained at GFN2-xTB and UB3LYP/def2-SVP levels.

The urea hydrolysis reaction starting with **1**′ is found to proceed via mechanism (v) (^**1**^**R** → ^**1**^**TS1A** → ^**1**^**Int1A** → ^**1**^**TS2A** → ^**1**^**PA**) calculated at both the GFN2-xTB and UB3LYP levels (Figure 3a).

Cartesian coordinates for all stationary points are presented in the Supporting Information. Figure 3a shows that the nucleophilic attack of the bridging hydroxide on the carbon atom of urea leads to the formation of the O(hydroxide)–C(urea) bond via ^**1**^**TS1A**, and afterward, H(hydroxide) is transferred not directly but via D363 to the NH_2_ group at ^**1**^**TS2A**. GFN2-xTB predicts that the proton transfer reaction (^**1**^**TS2A**) is the rate-determining step with an activation barrier of 11.5 kcal mol^–1^. On the other hand, UB3LYP indicates that the first nucleophilic attack (^**1**^**TS1A**) is rate-determining as it is characterized as a late transition state with an O(hydroxide)–C(urea) bond forming distance of 1.55 Å (Table S1) and requires a higher activation barrier than the second step (25.3 vs 23.8 kcal mol ^–1^). The resulting ^**1**^**PA** has a binding mode in line with the active site inhibited by acetohydroxamic acid.^17^

Figure 3b shows that urea hydrolysis starting from **2**′ agrees with mechanism (ii). The reaction is initiated by nucleophilic attack of O(hydroxide) on C(urea) via ^**2**^**TS1A** to form a tetrahedral intermediate ^**2**^**Int1A**. The O(hydroxide)···C(urea) distances at ^**2**^**TS1A** are 1.80 and 1.77 Å optimized at the GFN2-xTB and UB3LYP levels (Table S2). GFN2-xTB directly yields an intermediate ^**2**^**Int3A** in which the leaving amide nitrogen atom is triply protonated, while the reaction predicted by UB3LYP passes through an intermediate ^**2**^**Int2A** with the protonated D363 as well as ^**2**^**Int3A** (see also Table S3). The final reaction step is the collapse of ^**2**^**Int3A** involving dissociations of C–N and Ni2–N bonds, leading to ^**2**^**PA** with the same binding mode as ^**1**^**PA** (see also Tables S4 and S5). Unlike mechanism (v), this process is not spontaneous but requires ^**2**^**TS4A** barriers of 11.3 kcal mol^–1^ for GFN2-xTB and 17.6 kcal mol^–1^ for UB3LYP.

Based on the QM-only cluster calculations, it appears that mechanism (ii) is preferable for urea hydrolysis on the basis of the potential energy profiles obtained from GFN2-xTB and UB3LYP, though the calculated barrier heights both overestimate the experimental value of 5.7 kcal mol^–1^.^62,63^ Regarding the GFN2-xTB calculations, the highest activation barrier for mechanism (ii) (^**2**^**TS4A**) is slightly lower than that for mechanism (v) (^**1**^**TS2A**), and ^**2**^**PA** is more stable than ^**1**^**PA**. The results of UB3LYP clearly show the superiority of mechanism (ii) in terms of the computed lower barrier heights and larger exothermicity (Figure 3). The dissociation of W2 (^**1**^**R** → ^**1**^**TSB** → ^**1**^**PB**) also turns out much lower activation barriers of 4.9 and 1.5 kcal mol^–1^ obtained with GFN2-xTB and UB3LYP (Figure S5). This strongly supports that **2**′ can be predominantly formed by replacement of W2, as shown in Figure 1. We also find that the GFN2-xTB method can qualitatively reproduce the results of UB3LYP. As such, the use of GFN2-xTB for the QM region can be alternative to broken-symmetry DFT calculations with which simulations on the order of several tens of picoseconds are virtually prohibitive.

### 3.3 Free Energy Profiles Obtained from GFN2-xTB/MM Metadynamics Calculations

#### 3.3.1 Complex with Monodentate-Bound Urea (**1**)

**Figure 4:**
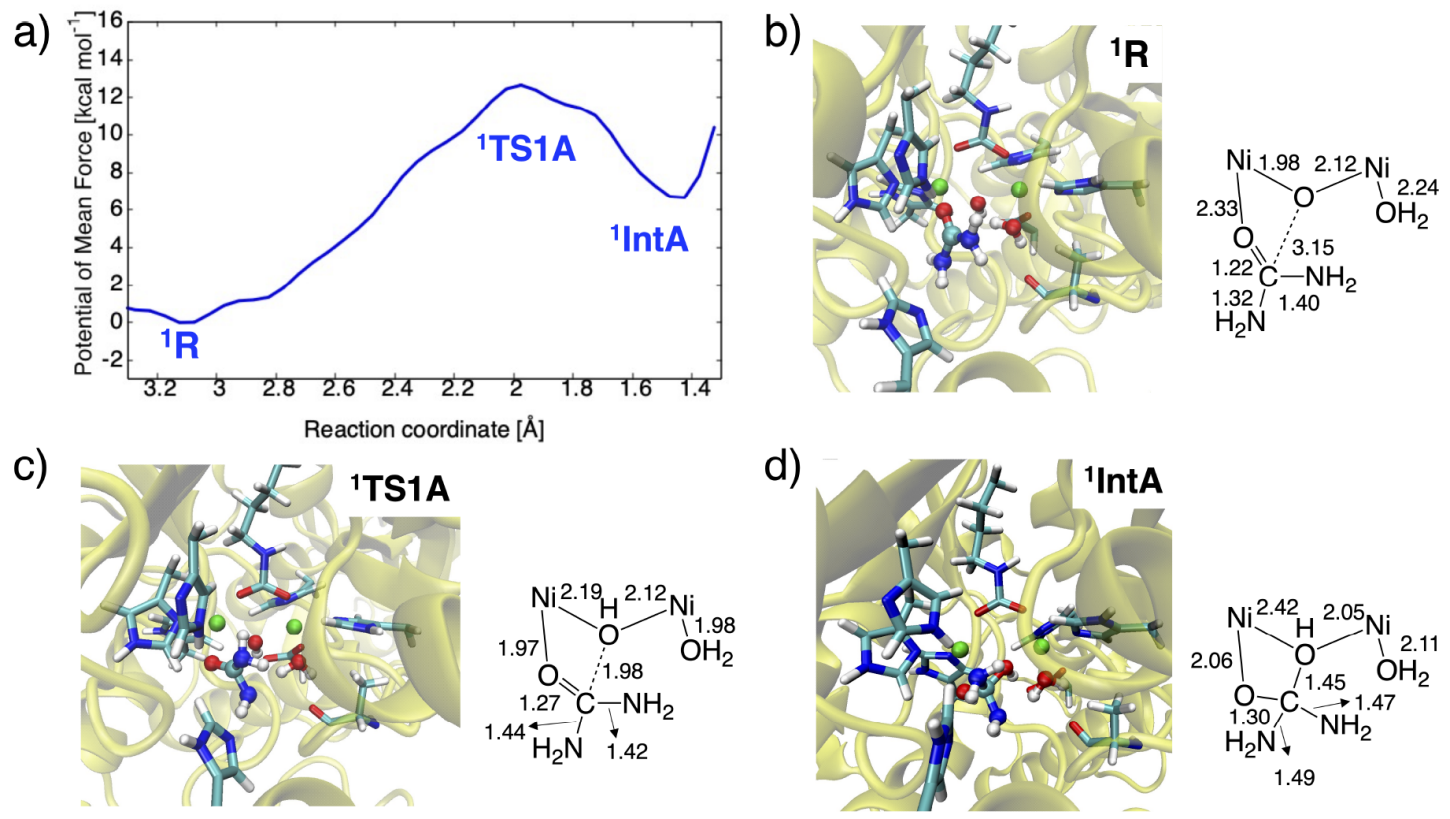
(a) One-dimensional potentials of mean force (1D-PMF) of the nucleophilic attack reaction and representative snapshots of the active site in (b) ^**1**^**R**, (c) ^**1**^**TS1A**, and (d) ^**1**^**IntA**, with key distances in Å.

Concerning the nucleophilic attack reaction in mechanism (v), the 1D-PMF is depicted in Figure 4 along the C(urea)···O(hydroxide) distance in a range from 1.30 to 3.40 Å. Also shown are the representative snapshots of ^**1**^**R**, ^**1**^**TS1A**, and ^**1**^**IntA**. During the nucleophilic attack, the bridging hydroxide ion and the carboxylic group of D363 keep forming a strong hydrogen-bonding interaction with an O···O distance of 2.82 ± 0.20 Å. It can be seen that ^**1**^**TS1A** for nucleophilic attack has a C–O bond forming distance of ca. 2.0 Å and requires a large activation barrier of 12.7 kcal mol^–1^. After ^**1**^**TS1A**, the C–O bond length is reduced to ca. 1.4 Å, leading to a tetrahedral intermediate ^**1**^**IntA** lying 6.7 kcal mol^–1^ above ^**1**^**R**. The high endergonicity of the step is analogous to the QM-only results. It suggests that the monodentate-bound substrate and intermediate at ^**1**^**TS1A** and ^**1**^**Int1A** are not stabilized by intramolecular interactions associated with a closed conformation of the mobile flap. Indeed, the flap of the initial X-ray crystal structure^24^ corresponds to the closed conformation in which specific active site residues (H222, H323, and A366) are close to urea, but hydrogen-bonding interactions between urea and these residues are not retained during the simulations. The O(urea)···N(His222), N(urea)···N(H323), and N(urea)···O(A366) distances are 4.21 ± 0.70, 3.42 ± 0.80, and 4.81 ± 0.62 Å, respectively.

**Figure 5:**
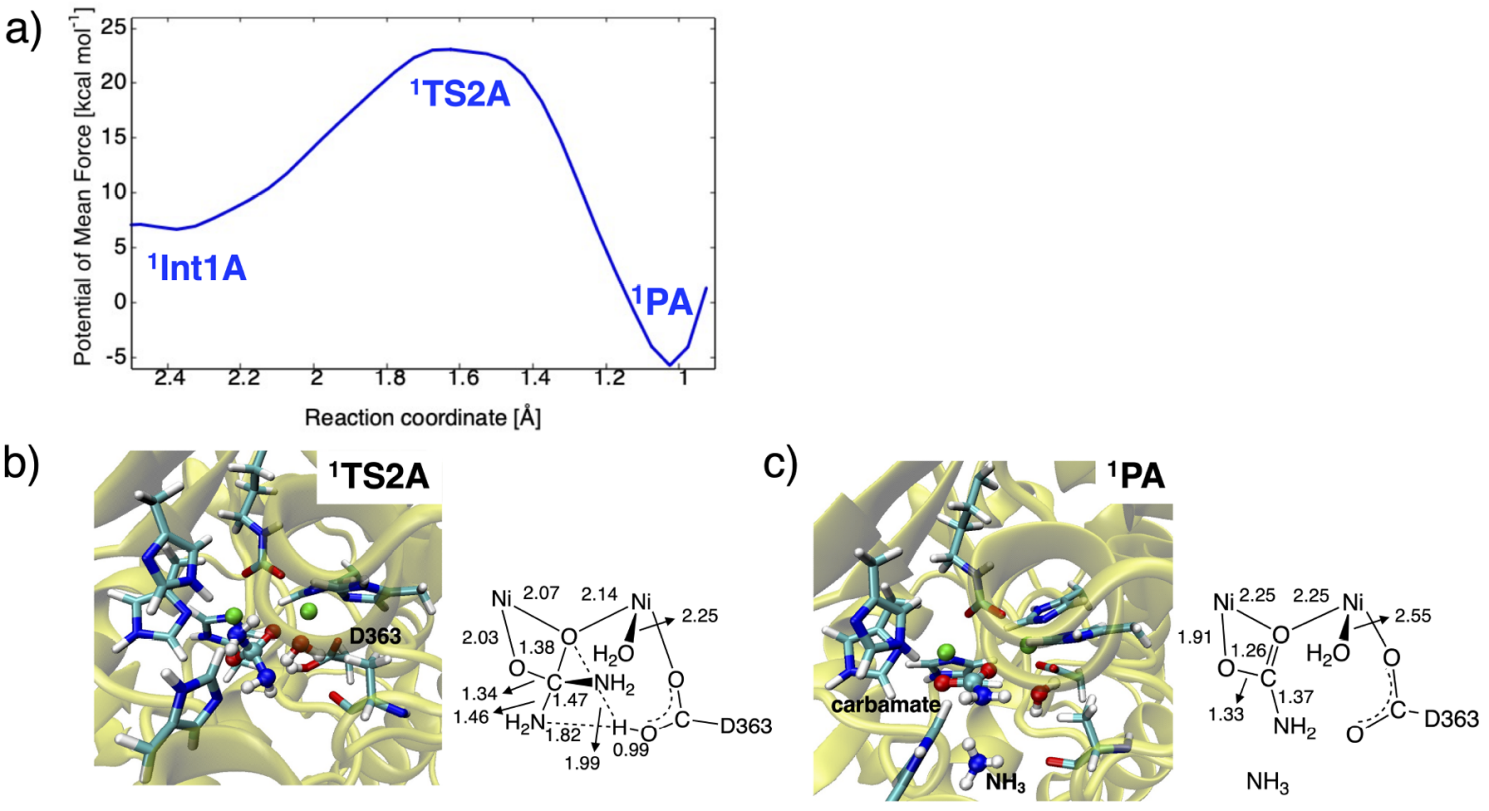
(a) One-dimensional potentials of mean force (1D-PMF) of the proton transfer reaction and representative snapshots of the active site in (b) ^**1**^**TS2A** and (c) ^**1**^**PA**, with key distances in Å.

Subsequently, the reaction undergoes a proton transfer from the bridging hydroxide ion to the leaving NH_2_ group of urea. Figure 5 shows the free energy profile along the distance between H(hydroxide) and N(urea) atoms, ranging from 0.90 to 2.60 Å. It also indicates that the proton is not transferred directly but via D363 to the NH_2_ group. The state with protonated D363 is found to be the rate-determining transition state (^**1**^**TS2A**). The computed activation and reaction free energies for ^**1**^**TS2A** and ^**1**^**PA** are +16.4 and −12.4 kcal mol^–1^ relative to ^**1**^**IntA**. The elimination reaction of the NH_2_ group is exergonic, possibly because a hydrogen bond is formed in ^**1**^**PA** between the carbamate product and H323, with an N(carbamate)···N(H323) distance of 3.03 ± 0.28 Å. The H222 and A366 residues do not form a stable hydrogen bonding with ^**1**^**PA** as in the case of the nucleophilic attack step. The overall reaction proves to be slightly exergonic by −5.7 kcal mol^–1^, whereas the proton transfer from the hydroxide ion seems unfavorable on the basis of its high free energy barrier of 23.1 kcal/mol relative to ^**1**^**R**. The activation barrier is also substantially higher than that of the QM-only model (Figure 3), emphasizing that the hydrolysis reaction of urea is more unlikely to proceed in the monodentate complex with the Ni2-coordinated W2.

**Figure 6:**
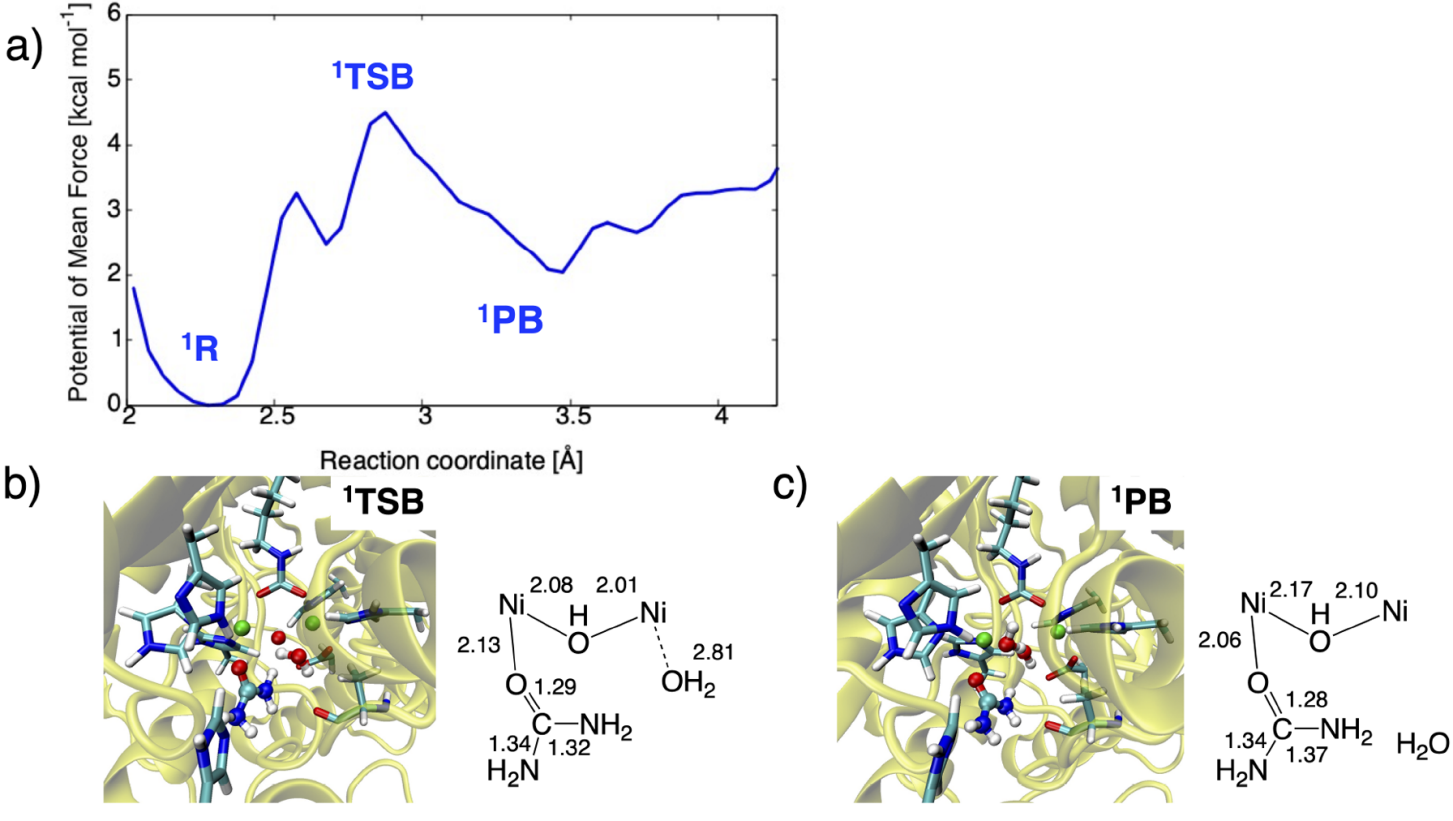
(a) One-dimensional potentials of mean force (1D-PMF) of the W2 dissociation and representative snapshots of the active site in (b) ^**1**^**TSB** and (c) ^**1**^**PB**, with key distances in Å.

Let us turn our attention to the dissociation of W2. Figure 6 displays its 1D-PMF obtained with the metadynamics simulations and the representative snapshots of the key states denoted as ^**1**^**R**, ^**1**^**TSB**, and ^**1**^**PB**. The Ni2···O(W2) distance ranging from 2.05 to 4.25 Å is used for the reaction coordinate. The activation and reaction free energies are calculated to be 4.5 and 2.0 kcal mol^–1^ comparable to the QM-only results, and they are considerably lower than those for the aforementioned urea hydrolysis reaction. The dissociated W2 may remain in the vicinity of the active site as suggested by the Ni2···O(W2) separation of ca. 3.5 Å in ^**1**^**PB**. It highlights that, compared with the urea hydrolysis, the W2 dissociation is likely to occur predominantly.

#### 3.3.2 Complex with Bidentate-Bound Urea (2)

**Figure 7:**
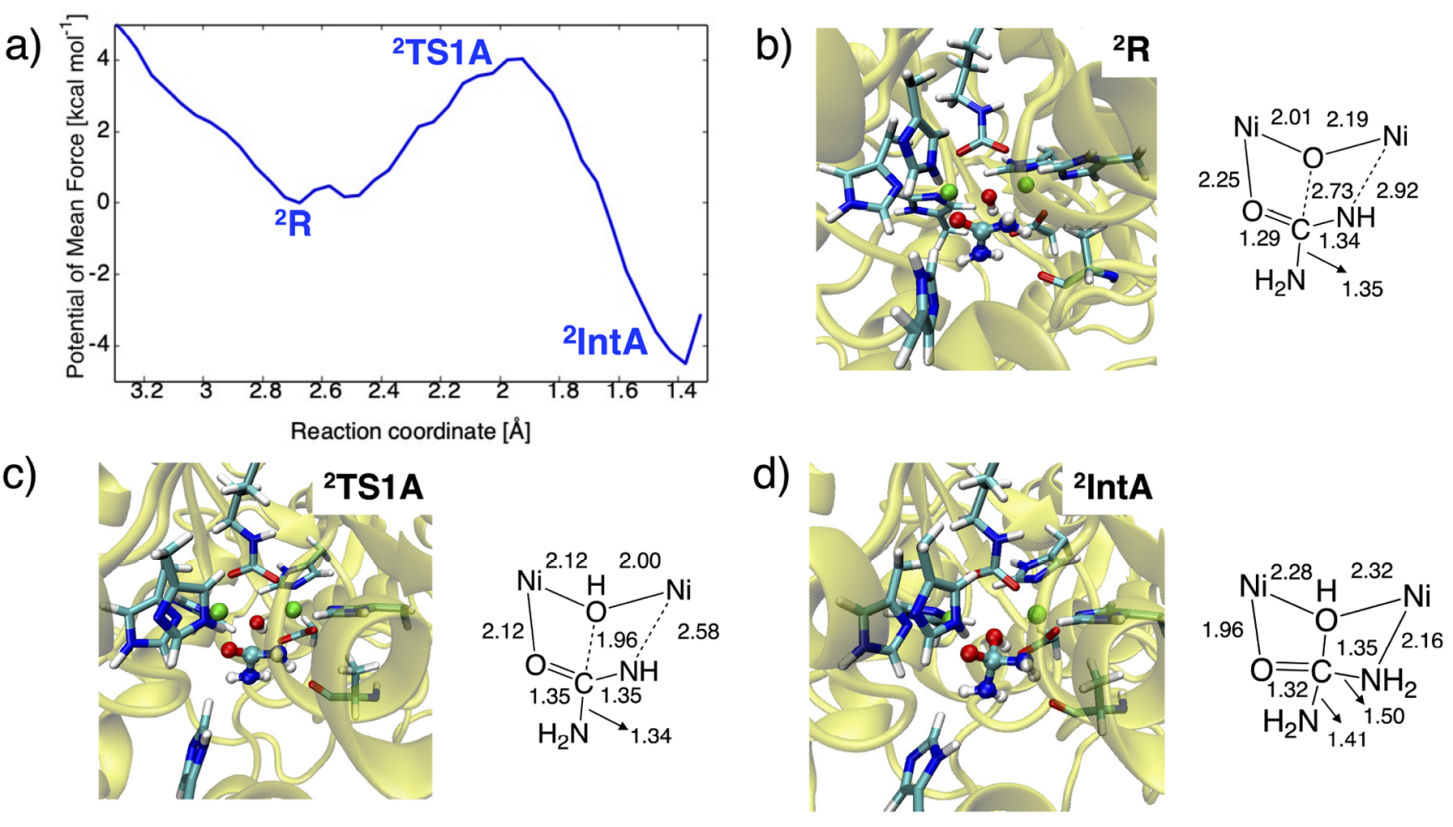
(a) One-dimensional potentials of mean force (1D-PMF) of the nucleophilic attack reaction and representative snapshots of the active site in (b) ^**2**^**R**, (c) ^**2**^**TS1A**, and (d) ^**2**^**IntA**, with key distances in Å.

The 1D-PMF corresponding to the nucleophilic attack is depicted along the C(urea)···O(hydroxide) distance in a range from 1.30 to 3.40 Å (Figure 7). The representative snapshots of ^**2**^**R**, ^**2**^**TS1A**, and ^**2**^**IntA** are also shown in Figure 7. Inspection of ^**2**^**R** reveals that urea can first bind to the dinickel center in a monodentate mode even in the absence of W2, having a longer Ni2···N(urea) separation of ca. 3.3 Å. In line with **1**, a strong hydrogen bonding is formed between the bridging hydroxide ion and the carboxylic group of D363, with an O···O distance of 2.74 ± 0.18 Å.

The nucleophilic attack step not only shortens the C(urea)–O(hydroxide) bond length but also increases the binding of one of the NH_2_ group of urea to Ni2. The C–O bond forming distance of ca. 2.0 Å at ^**2**^**TS1A** is similar to that at ^**1**^**TS1A**. The Ni2–N(urea) bond lengths at ^**2**^**TS1A** and ^**2**^**IntA** are reduced to 2.47 and 2.30 Å, respectively (Figure 7). The resulting activation free energy of 4.0 kcal mol^–1^ is close to experimentally measured values of ca. 5.7 kcal mol^–1^,^62,63^ and the reaction free energy of −4.5 kcal mol^–1^ is obviously lower compared with the result for mechanism (v) discussed above (6.7 kcal mol^–1^). These values are much lower than the corresponding values of +10.6 and −2.4 kcal mol^–1^ obtained from the QM-only model (Figure 3b). As such, the Ni2–N(urea) bond formation that occurs concomitantly with the nucleophilic attack can stabilize ^**2**^**TS1A** and ^**2**^**IntA**, causing a change of the binding mode from monodentate to bidentate. We also find that the bidentate complexes at ^**2**^**TS1A** and ^**2**^**IntA** form multiple hydrogen bonds with H222 as well as with A366 in which either or both NH_2_ groups are involved. The O(urea)···N(His222), the proximal N(urea)···O(A366), and the distal N(urea)···O(A366) distances are 3.01 ± 0.27, 3.17 ± 0.30, and 3.30 ± 0.31 Å, respectively. These features are consistent with the previous hypothesis^64^ that closure of the flap may be responsible for the coordination of the urea NH_2_ group to Ni2 and the formation of multiple hydrogen bonds with the nearby residues. In addition, it should be underlined that the active site can gain the benefits of the closed conformation only after replacement of W2, affording a coordinatively unsaturated Ni2 site.

**Figure 8:**
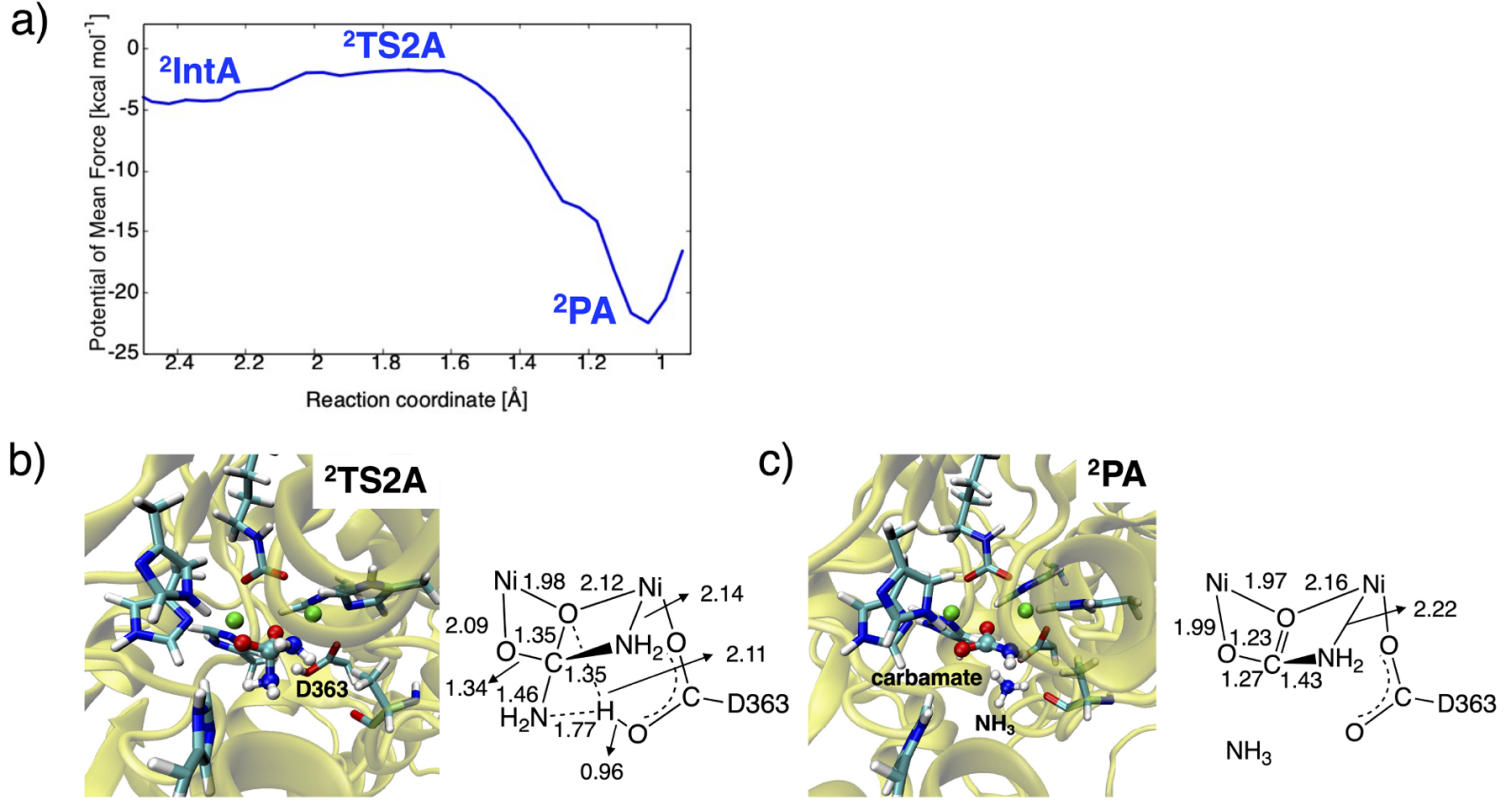
(a) One-dimensional potentials of mean force (1D-PMF) of the proton transfer reaction and representative snapshots of the active site in (b) ^**2**^**TS2A** and (c) ^**2**^**PA**, with key distances in Å.

Upon the formation of ^**2**^**IntA**, a proton transfer occurs from the bridging hydroxide ion to the leaving NH_2_ group only via ^**2**^**TS2A**, while the QM-only model in the absence of the protein environment involves ^**2**^**Int3A** and ^**2**^**TS4A**. Figure 8 shows the free energy profile along the distance between H(hydroxide) and N(urea) atoms in a range from 0.90 to 2.60 Å. Unlike the free energy landscape for mechanism (v) illustrated in Figure 5, the proton transfer step of mechanism (ii) turns out to be highly exergonic with ^**2**^**TS2A** and ^**2**^**PA** having free energies of −1.7 and −22.5 kcal mol^–1^ relative to ^**2**^**R**. In ^**2**^**PA**, the formed carbamate is coordinated to the metal center in a bidentate manner consistent with a urease complexed with diammonium phosphate.^23^ We see that the rigidity of the bidentate-bound tetrahedral intermediate and carbamate can maintain a hydrogen bond with H222 in the formation of ^**2**^**PA** continuing on the nucleophilic attack reaction, as the distance between the carbonyl oxygen atom and N(His222) decreases to 2.91 ± 0.18 Å. The generated NH_3_ also forms a weak hydrogen bond with D363 based on an N(NH_3_)···O(D363) distance of 3.06 ± 0.35 Å, whereas A366 and H323 seem not to play a significant role. Overall, the formation of the bidentate complex is crucial for catalysis because the subsequent reactions can proceed rapidly with benefits of the stabilization provided by the metal center and the surrounding protein.

## 4 Conclusions

The present study investigates the relationship between substrate binding and the catalytic mechanism of urease and explores possible reaction mechanisms with the use of QM-only cluster calculations and QM/MM free energy simulations. The QM region including the dinickel center and certain active site residues is treated by GFN2-xTB, which shows good performance in reproducing structures of both coordinatively unsaturated and saturated active-site model complexes. The QM(GFN2-xTB)/MM(CHARMM36) simulations provide evidence that the bridging hydroxide ion acts as both a nucleophile and a general acid, whereas a hydroxide ion that arises from the deprotonation of the Ni2-bound W2 can be excluded from a candidate for a nucleophile. The metadynamics simulations clearly demonstrate that urea hydrolysis via an active site complex without W2 (**2**) is much more plausible than via that with W2 (**1**), judging from the computed activation free energies for the rate-limiting step (4.0 vs 23.1 kcal mol^–1^) compared with the experimentally measured data (5.7 kcal mol^–1^). The high activation barriers for the hydrolysis process starting from **2** reflect the lack of stabilization via hydrogen-bonding interactions with nearby residues. We also reveal that the energetically favorable dissociation of W2 is a key step that triggers the binding of urea in an appropriate position prior to the hydrolysis process. The subsequent nucleophilic attack reaction involves a switch of the binding mode from monodentate to bidentate. In addition to the formation of the bidentate complex, comparison between the QM-only and QM/MM MD results demonstrates the importance of including the protein environment for the successive decomposition process. Indeed, the proton transfer reactions via D363 are significantly stabilized by forming multiple hydrogen bond interactions with the active site residues. Further studies are needed on the difference in reaction mechanism between urea and candidates for inhibitors to design more potent inhibitors. This work is in progress.

## Supporting Information Available

Figure S1, active site models for **1** and **2** excluding H222, H323, and A366 with key distances and spin density; Cartesian coordinates of these models obtained with the UB3LYP/def2-SVP level; the created topology and parameter files; Figure S2, RMSDs with respect to the initial conformations during a 10 ns classical MD simulation for **1** and **2**; Figure S3, overlay of smaller models (**1**, **2**, and **3**) optimized in the singlet and quintet states at GFN2-xTB with respect to the structure obtained with the broken-symmetry DFT method; Figure S4, structure and Cartesian coordinates of the QM region for **1** optimized in the open-shell singlet state at the UB3LYP/def2-SV(P) level; Figure S5, potential energy profile of the dissociation of W2; Cartesian coordinates for all stationary points of the QM-only cluster calculations optimized at the GFN2-xTB and UB3LYP levels; Tables S1–S5, key bond distance for all stationary points obtained from QM-only cluster and QM/MM metadynamics calculations; full citation for ref (59) (PDF)
