# Supplementary material for: QM/MM Molecular Dynamics Simulations Revealed Catalytic Mechanism of Urease
Source: J Phys Chem B. 2022 Mar 3;126(10):2087–97. doi: 10.1021/acs.jpcb.1c10200 (PMC8935366; doi:10.1021/acs.jpcb.1c10200)
Supplement: Supplementary file 1 — jp1c10200_si_001.pdf [file jp1c10200_si_001.pdf]

Supporting Information for

QM/MM Molecular Dynamics Simulations Revealed Catalytic Mechanism of Urease

Toru Saito and Yu Takano*

Department of Biomedical Information Sciences, Graduate School of Information Sciences, Hiroshima City University, 3-4-1 Ozuka-Higashi, Asa-Minami-Ku, Hiroshima 731-3194 Japan

* E-mail: tsaito@hiroshima-cu.ac.jp

Contents

Section 1: Active site models for **1** and **2** including Figure S1

Section 2: The created topology and parameter files

Section 3: Figure S2

Section 4: Figure S3

Section 5: Figure S4

Section 6: Figure S5

Section 7: Cartesian coordinates for all stationary points of the QM-only cluster calculations optimized at the GFN2-xTB and UB3LYP levels

Section 8: Tables S1-S5

Section 9: Full citation of ref. 59

Section 1: Active site models for 1 and 2 including Figure S1

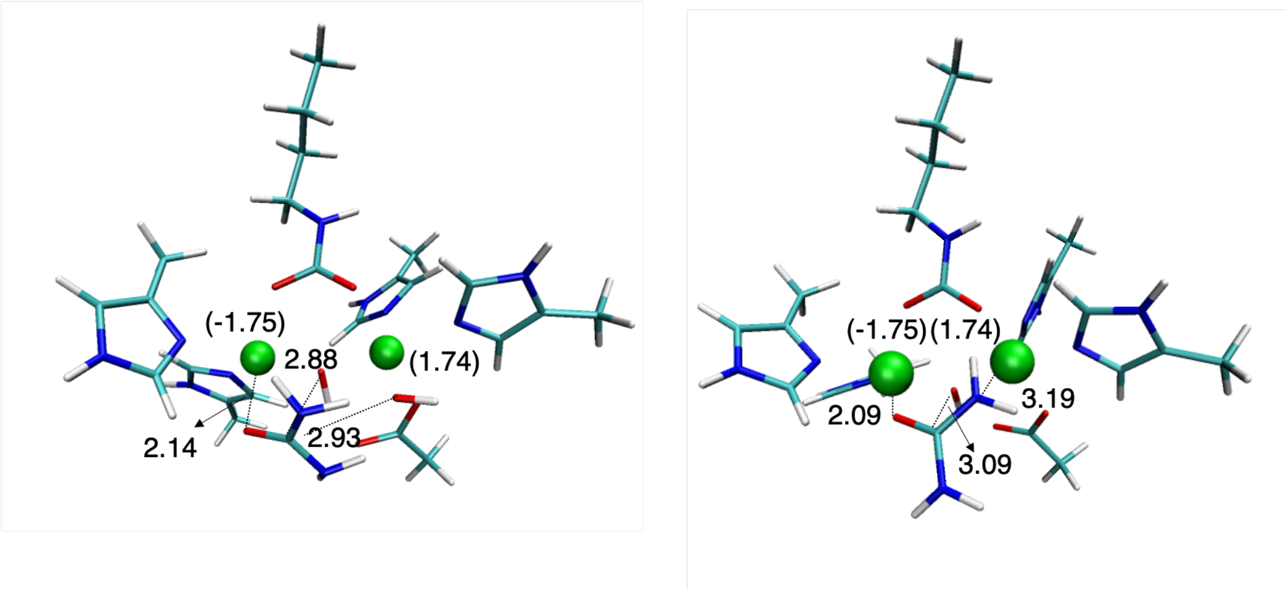

Figure S1. Active site models for **1** (left) and **2** (right). Key distances and spin density (in parentheses) are also provided.

Cartesian coordinates of active site model for **1**

C	26.69926544130992	78.16513881097063	69.52011229603326
H	26.23000399303864	78.99208627129124	70.08287223277334
H	27.46116731392963	78.60833928304713	68.85534375732014
H	25.92351657499272	77.71837884978281	68.88132842184100
N	28.40559107692007	77.30927710159757	71.19450406621500
H	28.97150224198706	78.14799044852032	71.23047653936442
C	27.27255432950809	77.12090833890738	70.41920936952845
C	28.63076594365100	76.16708660438860	71.90365281218763
H	29.46148834197464	75.99169753773418	72.58407245818624
N	27.71254503933166	75.26760606030764	71.63542844333712
C	26.86473277102303	75.83929873146990	70.71798753045752
H	26.00174384728914	75.30542731537572	70.32641800862820
C	22.00268222857216	73.12673799642180	71.91695667198590
H	21.55422436455580	73.48308489445535	70.97266847496991
H	21.49138611331622	72.18811458550276	72.19440292985250
H	21.76962563607600	73.86909868387380	72.69239309823026
N	24.09220005892263	72.11297381428201	70.89621941155517
H	23.61679245330260	71.52590589544229	70.22167135222371

C	23.48220619954186	72.95739408114679	71.81350892420015
C	25.44145902764247	72.19486383529038	71.07334031617485
H	26.18800118188088	71.64408206424140	70.50310217581885
N	25.72073859247037	73.03990982672828	72.03940528133603
C	24.52498311286554	73.52216292878551	72.51529354273586
H	24.51378436759259	74.22990380184665	73.34231277908643
C	29.39811151593693	72.88632320609290	64.66803719361361
H	30.18759247472464	73.40785080407392	64.10036278446650
H	29.19783414904174	71.93074686908822	64.15706103122663
H	28.48446822186184	73.50011162454271	64.59137519804111
C	29.80518038881159	72.67432587103700	66.12801648249034
H	29.02781345548503	72.09643288558181	66.65618196504037
H	30.71631376999226	72.05076863886092	66.17338462966561
C	30.06076632706420	73.98433392365254	66.88279751723579
H	30.85203080181776	74.55655845977196	66.36597652547138
H	29.15616648593602	74.62052512369095	66.83270750224250
C	30.47209771148199	73.81396513915359	68.34732756503530
H	31.37041400370995	73.17800353427924	68.39827449311841
H	30.77404478525091	74.79719982994656	68.76133660935321
N	29.42086578927839	73.20237115383391	69.13410200971107
H	28.46266646836809	73.34052251781473	68.83626824315790
C	29.55248747732590	72.84332562509290	70.47060223846789
O	28.52048297265824	72.43890694565538	71.05936853615327
O	30.73483434251520	72.93561836939941	70.94863926523237
C	33.74423839791481	72.40900443355572	70.14907703406838
H	32.69579800262824	72.66044592489388	69.94004092072647
H	34.26589044323072	73.35552484545161	70.37436126102854
H	34.21923463441676	71.97133806058318	69.25894082316977
N	32.96497277348850	71.57319587233960	72.40565302983909
C	33.80419500670587	71.46682154084078	71.30601798245448
C	33.31529111691354	70.61242134445303	73.24604025872422
H	32.84409931654498	70.45440808670155	74.21481575581365
N	34.33832541356531	69.88462830791855	72.74092843549758
H	34.76885877991454	69.08575104223368	73.18976904109708
C	34.66576659115690	70.41032370015192	71.50674487660751
H	35.45707065597249	69.99743881097825	70.88654319317635
C	33.14569204333493	77.72002849216787	75.20504858344101
H	33.79433694696286	77.64098289771007	76.09412143790128
H	33.54497199765268	78.53198878633280	74.57255374850219
H	32.14849006694180	78.02221365813202	75.55290562398535
N	34.15637743817595	75.82472969476585	73.87126282952252
H	35.10318009425126	76.18439848223466	73.87135557835251
C	33.05771927940051	76.42929635621174	74.46070452480492

C	33.74936891324277	74.66433270346432	73.29504463354810
H	34.41644053861980	73.97974182977789	72.77628428625928
N	32.45710141533738	74.49204939701448	73.47641747065279
C	32.00760862664590	75.57844118966342	74.19981733824746
H	30.97007149600027	75.65745856285560	74.51954295426565
C	26.48468968351211	75.87020967283514	76.29688028598561
H	25.82023229724205	75.18527938913154	76.85008297342854
H	27.08012299019130	76.44809775244789	77.01769567221385
H	25.83954335353428	76.54354903010945	75.71018171115990
C	27.38671118725344	75.05455669418532	75.35926466918946
O	26.79877456462294	74.53788189933562	74.36326756377260
O	28.60304146823724	74.95550765381962	75.65067346489980
Ni	31.24769605015371	72.90384028959666	72.92565583165391
Ni	27.66688980124555	73.36908616856861	72.83915125700740
O	29.57665161273441	73.78603878211457	73.38052448214198
H	29.42877510532157	74.11020273968721	74.29429370950285
C	30.15725400340548	71.44260850426279	74.95162568939003
O	31.34014244511781	71.88776166535304	74.80598246469158
N	29.30654403521715	71.92375502377651	75.88471934875508
H	29.53662979913504	72.86048866105770	76.20920905149015
H	28.33339232319844	71.85709591400536	75.51374938198745
N	29.66287872434529	70.44687118710692	74.16863421579158
H	30.24843533491476	70.22595650556416	73.37044749038904
H	28.63325968578580	70.63569015519919	73.95416213469159
O	27.41923024083461	71.68137636215810	73.94602951474889
H	26.48060991200418	71.47652664622815	74.02840478161437

Cartesian coordinates of active site model for **2**

C	26.57965881564844	78.20674380133261	69.06712994522208
H	25.78592298347727	78.90988330485132	69.37051417730946
H	27.36807101655118	78.79310504207211	68.56460840652763
H	26.14878765223843	77.52077229966581	68.32428693591324
N	27.64467423619624	78.02355132813813	71.36249278803460
H	27.73804465740895	79.01912154161209	71.52946152991187
C	27.10664265303042	77.43356940887510	70.23036236968773
C	28.00248292700164	77.04961919713649	72.23582862943074
H	28.42866161824774	77.21772019016517	73.22613047485157
N	27.72784166132395	75.86283057869508	71.72576660768306
C	27.17713815612283	76.08262961934398	70.48297069628140
H	26.85017691149164	75.26283499682697	69.84956659334009
C	22.21458028497993	73.14007864121133	72.59838516987357
H	21.62936170561968	73.49066791949507	71.73148876453389

H	21.83615660408955	72.14112496734487	72.87565863148677
H	22.00386774996470	73.81863648046441	73.43556167973270
N	24.25360346262958	72.39798782859278	71.28586221918223
H	23.75476706195149	71.81789941865622	70.62046558390307
C	23.67997857764814	73.12694394190923	72.31646545659531
C	25.59633851661317	72.59755897531790	71.31148401167708
H	26.30797210564534	72.15220529146382	70.61976152866265
N	25.91702475223714	73.41092845063619	72.30205824809593
C	24.73987522526041	73.74965741729416	72.93610182906890
H	24.75696387008711	74.41128155915183	73.79853671585771
C	29.26253164363943	73.00583010728657	64.82102102902755
H	30.09841764202234	73.40951372640815	64.22563240980412
H	28.89216222037485	72.10499901254282	64.30642693125036
H	28.45340623054147	73.75522543175175	64.79770708050586
C	29.70012363840461	72.69734428032594	66.25619340380621
H	28.85296887248141	72.25119443378107	66.80909562856993
H	30.48534379561133	71.91987914307728	66.24955852241695
C	30.22971491950235	73.92832814215640	67.00750081618401
H	31.08853711555129	74.34595036021884	66.45459476711639
H	29.46036094162188	74.72340592476817	67.01140078738725
C	30.67936219030770	73.66344505389732	68.44668421960900
H	31.43141128676960	72.85240286838462	68.46194326372191
H	31.18153011800461	74.55711861062362	68.85482859503264
N	29.55526060329619	73.32128213640362	69.30402963437382
H	28.64260392647614	73.18660029005049	68.88974376011461
C	29.63284102157981	73.13185586920957	70.65352881731408
O	28.53850283654553	72.84979599462483	71.26281775382392
O	30.77151980301192	73.24654632054609	71.19306965608645
C	33.99680342611087	72.96572613072442	70.90010554468940
H	33.02642483493361	73.31272833405677	70.52018444369440
H	34.44730971952418	73.80128396671851	71.46017089444079
H	34.65984616942543	72.73364131930788	70.05481751191097
N	32.79608863399157	71.64011850957411	72.70811285131151
C	33.81045116123288	71.76106882999090	71.76563458927099
C	32.93936154498524	70.45268391746716	73.27598176011719
H	32.27964789987772	70.04707690311081	74.03883311842058
N	33.99767026934857	69.79841082032519	72.75064865892558
H	34.31301762592285	68.87284892619327	73.01628773433340
C	34.56308838409735	70.60829651430366	71.78790192489531
H	35.42896353083908	70.30351446995941	71.20631086203838
C	33.34936228880482	78.10698035682132	73.73402507522452
H	33.65132578473407	78.49854335531391	74.71998389920748
H	34.12096975062667	78.40690257811295	73.00550198276763

H	32.41228343476845	78.60286757220533	73.44740935660781
N	34.15219455262505	75.74237676995469	74.12510410501466
H	35.08154203892229	75.99558214899754	74.44142561957615
C	33.15433157838957	76.62896652496983	73.75518395556882
C	33.67123616552394	74.48012794666862	74.01772395243367
H	34.25390026172836	73.58990652087886	74.24223953454580
N	32.42015339562913	74.50428872983019	73.59702187279642
C	32.08021718640369	75.82974224196448	73.43756646264545
H	31.08152871300092	76.10947483415460	73.11619447148739
C	26.62814286203611	76.36289861413121	76.34046134096587
H	26.04995078041426	75.56948308941006	76.83928902603580
H	27.21137827098826	76.92963389221539	77.07815159330532
H	25.89892156038423	77.03588108923276	75.85784359669242
C	27.53273955331623	75.76834093741064	75.26683696839974
O	26.95372663070010	74.95450161801588	74.46467368878911
O	28.72626512534719	76.11909684049211	75.20012751439269
Ni	31.13776952797179	72.93557686834687	73.14703806426783
Ni	27.82519210017419	74.12714204441247	72.83483272129851
O	29.54410877201295	73.93560772922122	73.72340233529675
H	29.57529844981908	74.65868196412337	74.38229873568390
C	29.14123787044101	70.92847837049034	74.32335586328264
O	30.32394015304973	71.23273565748836	74.05352955610837
N	28.83614982027243	70.42847467539467	75.55038567901867
H	29.55532332245084	70.47678014168466	76.26005003407842
H	27.88339798867257	70.27929432245620	75.85452008747156
N	28.15793684374614	71.01124750196226	73.40668222831938
H	28.35678697165574	71.53234121628975	72.53992459571521
H	27.18998979388170	70.86858261928063	73.66397382991759

Section 2: The created topology and parameter files

The atomic charges of the QM region for 1 on the basis of the ChelpG scheme were added to the topology file as follows:

```
RESI NI1              0.57 ! Ni(1) ion
GROUP
ATOM NI     NI        0.57
PATCHING FIRST NONE LAST NONE

RESI NI2              1.04 ! Ni(2) ion
GROUP
ATOM NI     NI        1.04
PATCHING FIRST NONE LAST NONE

RESI WBG             -0.35 ! bridging OH
GROUP
ATOM OW     OH1      -0.66
ATOM HW     H         0.31
BOND OW HW
PATCHING FIRST NONE LAST NONE

RESI WLG             -0.70 ! OH bound to Ni(2)
GROUP
ATOM OW     OH1      -1.08
ATOM HW     H         0.38
BOND OW HW
PATCHING FIRST NONE LAST NONE

RESI H01              0.05   ! neutral HIS, proton on ND1
GROUP
ATOM N      NH1      -0.47   !    |               HD1    HE1
ATOM HN     H         0.31   !  HN-N                |    /
ATOM CA     CT1       0.07   !    |   HB1      ND1--CE1
ATOM HA     HB1       0.09   !    |   |       /       ||
GROUP                        !  HA-CA--CB--CG         ||
ATOM CB     CT2      -0.45   !    |   |      \\       ||
ATOM HB1    HA2       0.18   !    |   HB2    CD2--NE2
ATOM HB2    HA2       0.18   !  O=C           |
ATOM ND1    NR1      -0.42   !    |           HD2
ATOM HD1    H         0.34
ATOM CG     CPH1      0.39
ATOM CE1    CPH2      0.20
ATOM HE1    HR1       0.03
ATOM NE2    NR2      -0.17
ATOM CD2    CPH1     -0.40
ATOM HD2    HR3       0.17
GROUP
ATOM C      C         0.51
ATOM O      O        -0.51
```

```
RESI H02             0.21   ! neutral HIS, proton on ND1
GROUP
ATOM N      NH1     -0.47
ATOM HN     H        0.31
ATOM CA     CT1      0.07
ATOM HA     HB1      0.09
GROUP
ATOM CB     CT2     -0.43
ATOM HB1    HA2      0.18
ATOM HB2    HA2      0.18
ATOM ND1    NR1     -0.28
ATOM HD1    H        0.32
ATOM CG     CPH1     0.28
ATOM CE1    CPH2    -0.12
ATOM HE1    HR1      0.17
ATOM NE2    NR2      0.06
ATOM CD2    CPH1    -0.35
ATOM HD2    HR3      0.20
GROUP
ATOM C      C        0.51
ATOM O      O       -0.51

RESI KCX            -0.66
GROUP
ATOM N      NH1     -0.47   !     |
ATOM HN     H        0.31   !  HN-N
ATOM CA     CT1      0.07   !     |   HB1 HG1 HD1 HE1 HZ1
ATOM HA     HB1      0.09   !     |   |   |   |   |   |
GROUP                       !  HA-CA--CB--CG--CD--CE--NZ--CF=O1
ATOM CB     CT2     -0.31   !     |   |   |   |   |       |
ATOM HB1    HA2      0.09   !     |   HB2 HG2 HD2 HE2     O2
ATOM HB2    HA2      0.09   !   O=C
ATOM CG     CT2      0.27
ATOM HG1    HA2     -0.04
ATOM HG2    HA2     -0.04
ATOM CD     CT2     -0.15
ATOM HD1    HA2      0.02
ATOM HD2    HA2      0.04
ATOM CE     CT2      0.20
ATOM HE1    HA2      0.01
ATOM HE2    HA2      0.00
ATOM NZ     NH1     -0.68
ATOM HZ1    H        0.31
ATOM CF     CC       1.00
ATOM O1     OC      -0.78
ATOM O2     OC      -0.69
GROUP
ATOM C      C        0.51
ATOM O      O       -0.51
```

```
RESI H03              0.26   ! neutral His, proton on NE2
GROUP
ATOM N      NH1      -0.47   !     |                   HE1
ATOM HN     H         0.31   ! HN-N                  --  /
ATOM CA     CT1       0.07   !     |   HB1     ND1--CE1
ATOM HA     HB1       0.09   !     |    |     /        |
GROUP                        !  HA-CA--CB--CG          |
ATOM CB     CT2      -0.49   !     |    |    \\        |
ATOM HB1    HA2       0.20   !     |   HB2     CD2--NE2
ATOM HB2    HA2       0.20   !  O=C              |     |
ATOM ND1    NR2      -0.10   !     |            HD2   HE2
ATOM CG     CPH1      0.30
ATOM CE1    CPH2     -0.14
ATOM HE1    HR1       0.19
ATOM NE2    NR1      -0.11
ATOM HE2    H         0.30
ATOM CD2    CPH1     -0.36
ATOM HD2    HR3       0.19
GROUP
ATOM C      C         0.51
ATOM O      O        -0.51

RESI H04              0.26   ! neutral HIS, proton on ND1
GROUP
ATOM N      NH1      -0.47
ATOM HN     H         0.31
ATOM CA     CT1       0.07
ATOM HA     HB1       0.09
GROUP
ATOM CB     CT2      -0.42
ATOM HB1    HA2       0.19
ATOM HB2    HA2       0.19
ATOM ND1    NR1      -0.32
ATOM HD1    H         0.33
ATOM CG     CPH1      0.28
ATOM CE1    CPH2     -0.02
ATOM HE1    HR1       0.15
ATOM NE2    NR2      -0.05
ATOM CD2    CPH1     -0.27
ATOM HD2    HR3       0.20
GROUP
ATOM C      C         0.51
ATOM O      O        -0.51

RESI A01             -0.71
GROUP
ATOM N      NH1      -0.47   !     |
ATOM HN     H         0.31   !  HN-N
ATOM CA     CT1       0.07   !     |   HB1   OD1
ATOM HA     HB1       0.09   !     |    |   //
GROUP                        !  HA-CA--CB--CG
ATOM CB     CT2A     -0.45   !     |    |   \
ATOM HB1    HA2       0.15   !     |   HB2   OD2(-)
```

```
ATOM HB2    HA2        0.15   !     O=C
ATOM CG     CC         0.91   !       |
ATOM OD1    OC        -0.73
ATOM OD2    OC        -0.74
GROUP
ATOM C      C          0.51
ATOM O      O         -0.51
```

The atomic charges of the QM region for 2 on the basis of the ChelpG scheme were added to the topology file as follows:

```
RESI NI1              0.63 ! Ni(1) ion
GROUP
ATOM NI     NI         0.63
PATCHING FIRST NONE LAST NONE

RESI NI2              0.65 ! Ni(2) ion
GROUP
ATOM NI     NI         0.65
PATCHING FIRST NONE LAST NONE

RESI WBG             -0.41 ! bridging OH
GROUP
ATOM OW     OH1       -0.84
ATOM HW     H          0.43
BOND OW HW
PATCHING FIRST NONE LAST NONE

RESI H01              0.26   ! neutral HIS, proton on ND1
GROUP
ATOM N      NH1       -0.47
ATOM HN     H          0.31
ATOM CA     CT1        0.07
ATOM HA     HB1        0.09
GROUP
ATOM CB     CT2       -0.45
ATOM HB1    HA2        0.20
ATOM HB2    HA2        0.20
ATOM ND1    NR1       -0.32
ATOM HD1    H          0.34
ATOM CG     CPH1       0.31
ATOM CE1    CPH2      -0.01
ATOM HE1    HR1        0.17
ATOM NE2    NR2       -0.03
ATOM CD2    CPH1      -0.31
ATOM HD2    HR3        0.16
GROUP
ATOM C      C          0.51
ATOM O      O         -0.51
```

```
RESI H02              0.29
GROUP
ATOM N      NH1     -0.47
ATOM HN     H        0.31
ATOM CA     CT1      0.07
ATOM HA     HB1      0.09
GROUP
ATOM CB     CT2     -0.44
ATOM HB1    HA2      0.20
ATOM HB2    HA2      0.20
ATOM ND1    NR1     -0.35
ATOM HD1    H        0.35
ATOM CG     CPH1     0.36
ATOM CE1    CPH2    -0.05
ATOM HE1    HR1      0.16
ATOM NE2    NR2      0.07
ATOM CD2    CPH1    -0.44
ATOM HD2    HR3      0.23
GROUP
ATOM C      C        0.51
ATOM O      O       -0.51

RESI KCX             -0.47
GROUP
ATOM N      NH1     -0.47
ATOM HN     H        0.31
ATOM CA     CT1      0.07
ATOM HA     HB1      0.09
GROUP
ATOM CB     CT2     -0.33
ATOM HB1    HA2      0.12
ATOM HB2    HA2      0.12
ATOM CG     CT2      0.22
ATOM HG1    HA2     -0.04
ATOM HG2    HA2     -0.02
ATOM CD     CT2     -0.14
ATOM HD1    HA2      0.05
ATOM HD2    HA2      0.05
ATOM CE     CT2      0.06
ATOM HE1    HA2      0.07
ATOM HE2    HA2      0.07
ATOM NZ     NH1     -0.58
ATOM HZ1    H        0.33
ATOM CF     CC       0.91
ATOM O1     OC      -0.81
ATOM O2     OC      -0.55
GROUP
ATOM C      C        0.51
ATOM O      O       -0.51

RESI H03              0.23   ! neutral His, proton on NE2
GROUP
ATOM N      NH1     -0.47
```

```
ATOM HN     H        0.31
ATOM CA     CT1      0.07
ATOM HA     HB1      0.09
GROUP
ATOM CB     CT2     -0.48
ATOM HB1    HA2      0.20
ATOM HB2    HA2      0.20
ATOM ND1    NR2     -0.12
ATOM CG     CPH1     0.30
ATOM CE1    CPH2    -0.10
ATOM HE1    HR1      0.16
ATOM NE2    NR1     -0.10
ATOM HE2    H        0.31
ATOM CD2    CPH1    -0.34
ATOM HD2    HR3      0.20
GROUP
ATOM C      C        0.51
ATOM O      O       -0.51

RESI H04             0.24   ! neutral HIS, proton on ND1
GROUP
ATOM N      NH1     -0.47
ATOM HN     H        0.31
ATOM CA     CT1      0.07
ATOM HA     HB1      0.09
GROUP
ATOM CB     CT2     -0.44
ATOM HB1    HA2      0.20
ATOM HB2    HA2      0.20
ATOM ND1    NR1     -0.26
ATOM HD1    H        0.33
ATOM CG     CPH1     0.25
ATOM CE1    CPH2    -0.03
ATOM HE1    HR1      0.14
ATOM NE2    NR2     -0.09
ATOM CD2    CPH1    -0.20
ATOM HD2    HR3      0.14
GROUP
ATOM C      C        0.51
ATOM O      O       -0.51

RESI A01            -0.64
GROUP
ATOM N      NH1     -0.47
ATOM HN     H        0.31
ATOM CA     CT1      0.07
ATOM HA     HB1      0.09
GROUP
ATOM CB     CT2A    -0.50
ATOM HB1    HA2      0.18
ATOM HB2    HA2      0.18
ATOM CG     CC       0.85
ATOM OD1    OC      -0.67
```

```
ATOM OD2    OC          -0.68
GROUP
ATOM C       C            0.51
ATOM O       O           -0.51
```

Added parameters of KCX added to the parameter file.

```
BONDS
!
!V(bond) = Kb(b - b0)**2
!
!Kb: kcal/mole/A**2
!b0: A
!
!atom type Kb                  b0
CC NH1          400.000         1.3300

V(angle) = Ktheta(Theta - Theta0)**2
!
!V(Urey-Bradley) = Kub(S - S0)**2
!
!Ktheta: kcal/mole/rad**2
!Theta0: degrees
!Kub: kcal/mole/A**2 (Urey-Bradley)
!S0: A
!
!atom types         Ktheta       Theta0      Kub          S0
CC NH1 CT2             90.000      122.0000
NH1 CC OC              90.000      117.0000
CC NH1 H               90.000      117.0000

DIHEDRALS
!
!V(dihedral) = Kchi(1 + cos(n(chi) - delta))
!
!Kchi: kcal/mole
!n: multiplicity
!delta: degrees
!
!atom types                        Kchi        n      delta
!
CT2    CT2 NH1    CC              0.0000    1         0.00
CT2    NH1 CC     OC              0.0000    1         0.00
HA2 CT2 NH1 CC                    0.0000    1         0.00
H NH1 CC OC                       0.0000    1         0.00
```

Added atomic charges and parameters of urea for 1 (2) added to the topology and parameter files.

```
RESI URE          0.02 (0.22)
GROUP
ATOM C1         CG2O6     1.00 (0.95)
ATOM O1         OG2D1    -0.70 (-0.58)
ATOM N1         NG2S2    -0.91 (-0.88)
ATOM H1         HGP1      0.39 (0.39)
ATOM H2         HGP1      0.42 (0.40)
ATOM N2         NG2S2    -0.95 (-0.89)
ATOM H3         HGP1      0.37 (0.45)
ATOM H4         HGP1      0.40 (0.38)

PATCHING FIRST NONE LAST NONE
                       ! Bond order
BOND H3     N2     ! 1
BOND O1     C1     ! 2
BOND N2     C1     ! 1
BOND N2     H4     ! 1
BOND C1     N1     ! 1
BOND N1     H1     ! 1
BOND N1     H2     ! 1
IMPR C1       N2       N1       O1

END

read param card flex append
* Parameters generated by analogy by
* CHARMM General Force Field (CGenFF) program version 2.4.0
*

! Penalties lower than 10 indicate the analogy is fair; penalties between 10
! and 50 mean some basic validation is recommended; penalties higher than
! 50 indicate poor analogy and mandate extensive validation/optimization.

BONDS
CG2O6   NG2S2    430.00      1.3600 ! UREA, Urea. Uses a slack parameter from PROT from
NG2S2   CT3, neutral glycine, adm jr. ==> re-optimize
CG2O6   OG2D1    650.00      1.2300 ! UREA, Urea. Uses a slack parameter from PROT adm
jr. 4/10/91, acetamide ==> re-optimize
NG2S2   HGP1     480.00      1.0000 ! PROT adm jr. 8/13/90 acetamide geometry and
vibrations

ANGLES
NG2S2   CG2O6   NG2S2     70.00     115.00 ! UREA, Urea
NG2S2   CG2O6   OG2D1     75.00     122.50     50.00     2.37000 ! UREA, Urea. Uses a
slack parameter from PROT adm jr. 4/10/91, acetamide update ==> re-optimize
CG2O6   NG2S2   HGP1      50.00     120.00 ! PROT his, adm jr. 8/13/90  geometry and
vibrations NOW UREA ==> re-optimize???
HGP1    NG2S2   HGP1      23.00     120.00 ! PROT adm jr. 8/13/90  geometry and
```

vibrations

DIHEDRALS
NG2S2 CG2O6 NG2S2 HGP1 1.5000 2 180.00 ! UREA, Urea
OG2D1 CG2O6 NG2S2 HGP1 1.4000 2 180.00 ! PROT adm jr. 4/10/91, acetamide update NOW UREA ==> re-optimize???

IMPROPERS
CG2O6 NG2S2 NG2S2 OG2D1 80.0000 0 0.00 ! UREA, Urea

END
RETURN

Section 3: Figure S2

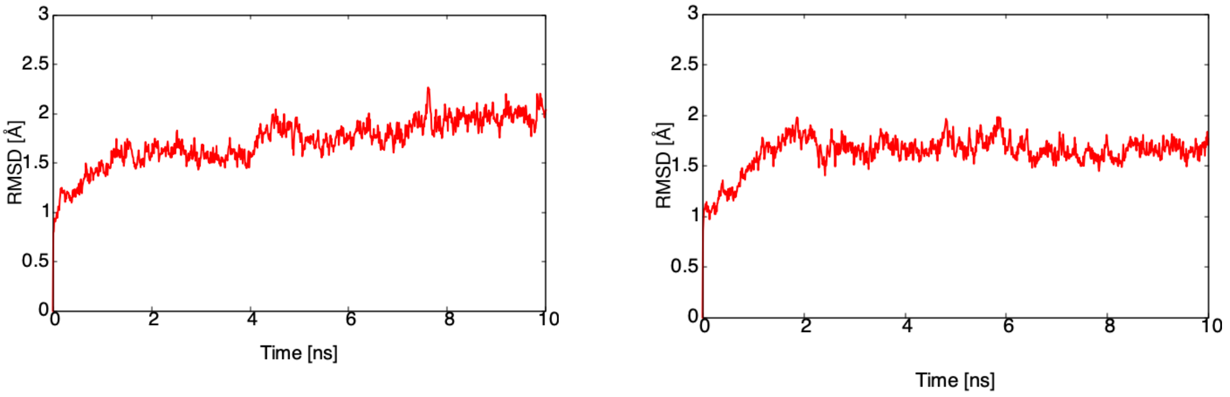

Figure S2. RMSDs with respect to the initial conformations during a 10 ns classical MD simulation for **1** (left) and **2** (right).

Section 4: Figure S3

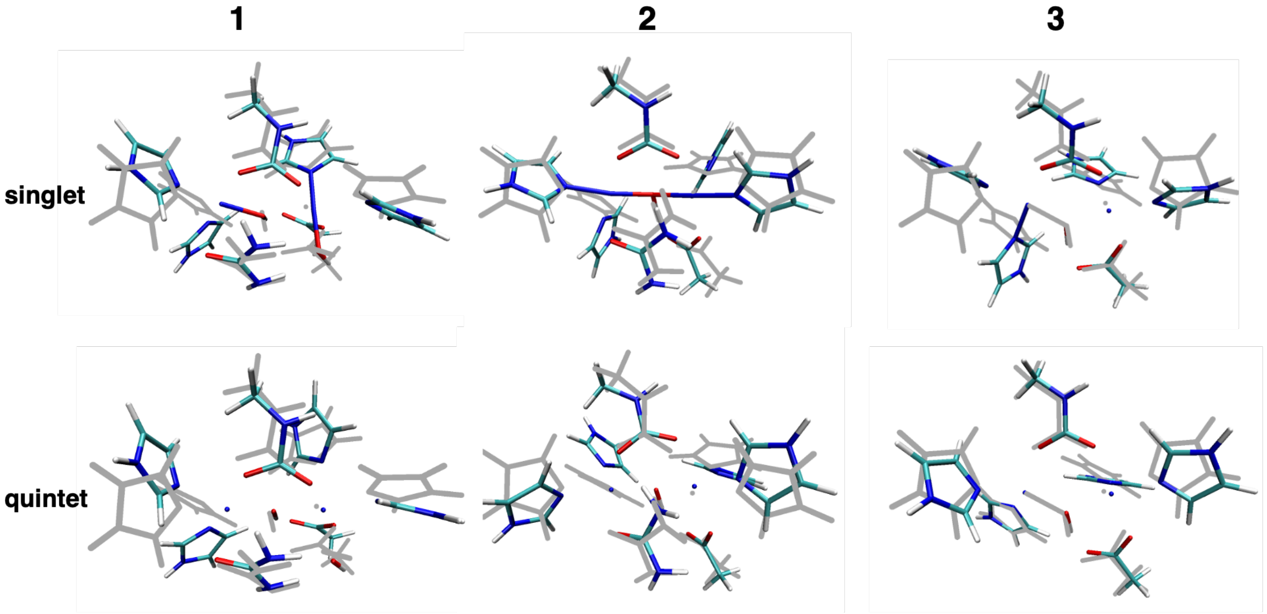

Figure S3. Overlay of **1**, **2**, and **3** optimized in the singlet and quintet states at GFN2-xTB with respect to the structure obtained with the UB3LYP/def2-SVP method (black).

Section 5: Figure S4

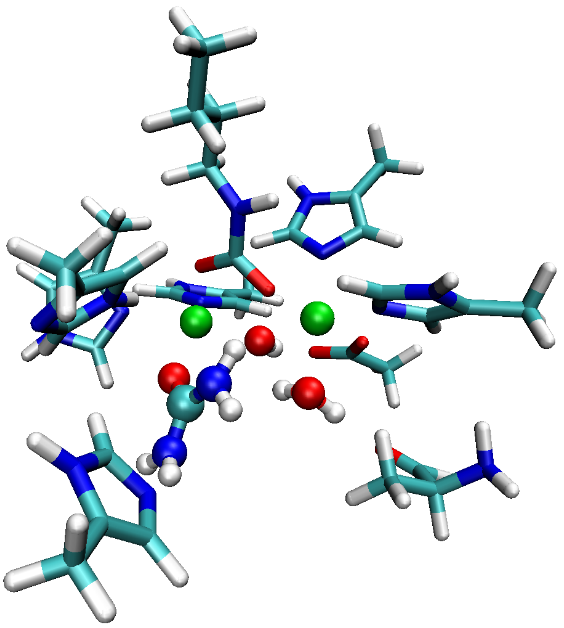

Figure S4. UB3LYP/def2-SV(P) optimized structure of the QM region of **1**.

Cartesian coordinates of the above complex

C	3.769886	-9.636761	-1.103189
H	4.118506	-8.654593	-0.731767
H	3.981932	-9.678719	-2.188038
N	5.768838	-10.994172	-0.333209
H	6.474402	-10.424904	-0.787720
C	4.401076	-10.770118	-0.362888
C	6.007906	-12.090812	0.432880
H	7.000565	-12.487966	0.633746
N	4.877238	-12.586617	0.892485
C	3.871594	-11.779683	0.408742
H	2.828097	-11.962377	0.656314
C	-1.059275	-14.510785	1.752462
H	-1.572710	-15.416064	2.127209
H	-1.259203	-13.694205	2.464857
N	0.975300	-15.702935	0.815685
H	0.463359	-16.377365	0.257590
C	0.414363	-14.717225	1.614651
C	2.328825	-15.598689	0.891614
H	3.031499	-16.248483	0.372280
N	2.667070	-14.629317	1.716086

C	1.494345	-14.073824	2.179494
H	1.524725	-13.256655	2.898446
C	5.762754	-16.924732	-5.352553
H	4.789385	-16.428441	-5.519128
H	5.628206	-18.000915	-5.557503
C	6.273872	-16.689368	-3.928709
H	5.566400	-17.136624	-3.204481
H	7.231493	-17.224802	-3.782373
C	6.480041	-15.204619	-3.598216
H	7.178913	-14.760956	-4.332421
H	5.524603	-14.655557	-3.726324
C	7.038463	-14.927108	-2.199039
H	7.988111	-15.471590	-2.061356
H	7.275478	-13.847848	-2.103349
N	6.109932	-15.340683	-1.158878
H	5.125805	-15.402405	-1.396786
C	6.373633	-15.335437	0.187480
O	5.407223	-15.602299	0.965382
O	7.576309	-15.090675	0.543890
C	7.533237	-21.033420	-1.450998
H	7.090936	-21.836233	-0.829568
H	6.730443	-20.619560	-2.085420
N	9.233365	-20.233328	0.223760
C	8.158928	-19.969267	-0.600695
C	9.521211	-19.091009	0.813354
H	10.323083	-18.929498	1.536171
N	8.686408	-18.090642	0.419281
H	8.655058	-17.139626	0.786619
C	7.802904	-18.639364	-0.483182
H	7.012719	-18.048936	-0.941734
C	10.650300	-14.603566	-0.408475
H	9.925452	-15.230677	-0.954395
H	10.150250	-13.640266	-0.211871
N	10.223660	-15.438640	1.948788
C	11.070550	-15.267440	0.862953
C	10.910175	-16.094873	2.869763
H	10.520137	-16.401117	3.837784
N	12.165883	-16.352240	2.437841
H	12.884588	-16.845883	2.956519
C	12.290184	-15.831495	1.165717
H	13.206357	-15.918569	0.586233
C	9.165272	-9.211955	3.889035
H	8.123780	-9.081972	4.223250

H	9.820125	-9.041898	4.763723
N	10.544710	-11.086226	2.870318
H	11.429220	-10.590115	2.857274
C	9.336014	-10.584344	3.326593
C	10.354466	-12.365563	2.469246
H	11.153262	-12.991736	2.076573
N	9.088292	-12.720618	2.628191
C	8.444584	-11.618158	3.165092
H	7.396712	-11.635223	3.465837
C	5.893563	-24.522170	3.014228
H	6.754020	-25.207465	3.124634
H	5.127808	-24.830807	3.744452
C	5.862015	-22.168006	4.180077
H	5.124082	-22.304628	4.970539
C	6.278614	-23.098154	3.250298
N	6.519785	-20.971908	3.999578
H	6.987110	-19.229840	4.647872
N	7.219594	-22.424470	2.490352
H	7.754203	-22.791587	1.709168
C	7.329059	-21.160856	2.976745
H	8.007706	-20.429349	2.540814
C	3.563727	-11.034992	5.228800
H	3.165645	-11.630898	6.070230
H	4.161970	-10.205624	5.636492
C	4.424471	-11.952276	4.365898
O	3.782965	-12.748390	3.600254
O	5.666881	-11.883078	4.467042
N	-0.152885	-15.862915	5.418265
H	0.059925	-15.679557	4.434078
C	1.062935	-16.208334	6.152281
H	0.735731	-16.580989	7.143227
C	1.982038	-17.241119	5.496113
H	2.341850	-16.887857	4.515356
H	1.435677	-18.189599	5.349638
H	2.873603	-17.447415	6.113744
C	1.751975	-14.882106	6.454983
O	2.863904	-14.565433	6.097591
Ni	8.216989	-14.698364	2.438009
Ni	4.624846	-14.123054	2.340709
O	6.502766	-14.091075	3.170081
H	6.438185	-13.280297	3.734581
O	4.532502	-15.541181	3.981795
H	3.961192	-15.193285	4.702835

C	7.297337	-17.456458	3.607696
O	8.115051	-16.515232	3.416448
N	7.473621	-18.314342	4.638727
H	8.314979	-18.200964	5.190177
H	5.451583	-15.189504	4.129425
N	6.253648	-17.685285	2.783739
H	5.946073	-16.922401	2.165087
H	5.530541	-18.323889	3.099216
H	2.676526	-9.669366	-0.976142
H	-1.531604	-14.236304	0.789809
H	6.469285	-16.527663	-6.104370
H	8.282162	-21.508683	-2.108912
H	11.523030	-14.427801	-1.060280
H	9.387786	-8.423719	3.145049
H	5.472766	-24.676304	2.002376
H	2.703557	-10.644460	4.658336
H	-0.849008	-16.607178	5.463617
H	1.113693	-14.166060	7.029500

Section 6: Figure S5

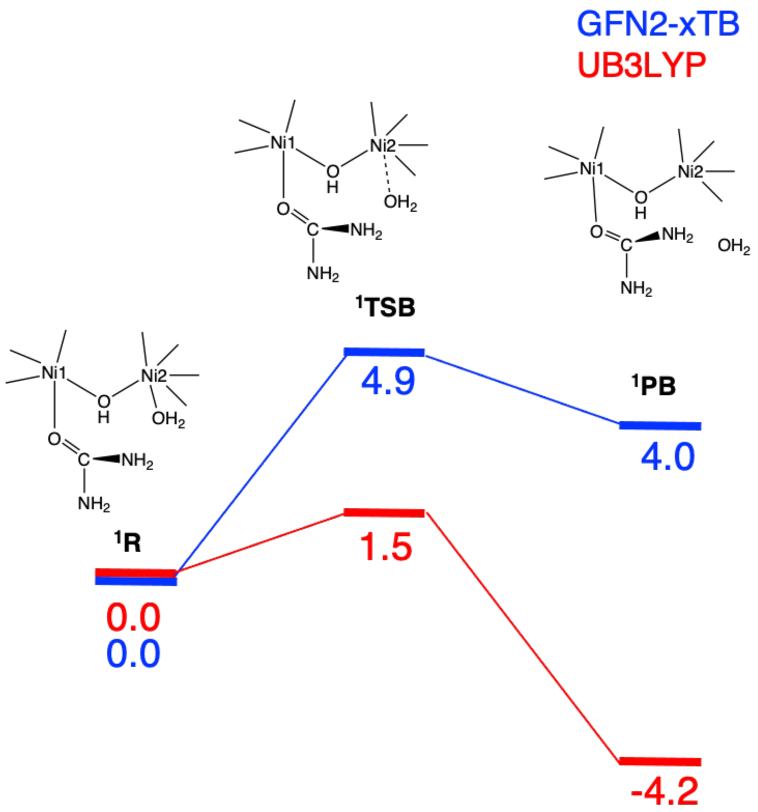

Figure S5. Potential energy profiles (in kcal mol^{-1}) for W2 dissociation starting from **1'** obtained with GFN2-xTB and UB3LYP.

Section 7: Cartesian coordinates for all stationary points of the QM-only cluster calculations optimized at the GFN2-xTB and UB3LYP levels

GFN2-xTB

[1]R

N 5.638624 -10.653945 0.589920
H 6.339946 -9.939528 0.484372
C 4.289828 -10.470875 0.490990
C 5.849358 -11.936951 0.930741
H 6.818480 -12.374539 1.077996
N 4.710855 -12.580582 1.041298
C 3.725454 -11.678957 0.770950
H 2.690741 -11.954274 0.798777
N 1.108107 -16.053381 0.775734
H 0.638615 -16.742480 0.210232
C 0.502492 -15.141353 1.590631
C 2.437172 -15.861639 0.871884
H 3.175168 -16.430341 0.342357
N 2.705130 -14.880494 1.699064
C 1.509416 -14.418614 2.158351
H 1.464990 -13.612468 2.862291
C 7.406716 -14.366145 -2.141524
H 7.625443 -15.156024 -2.864643
H 8.301505 -14.157122 -1.558950
N 6.376228 -14.770619 -1.221442
H 5.451065 -14.946719 -1.577868
C 6.571926 -15.000827 0.109107
O 5.566361 -15.407459 0.751728
O 7.734696 -14.818195 0.559622
N 10.257908 -15.473419 2.231206
C 10.592777 -16.155629 1.099495
C 11.062518 -15.903674 3.173931
H 11.062944 -15.564010 4.190531
N 11.911007 -16.832101 2.695062
H 12.621577 -17.322410 3.214440
C 11.622381 -17.005924 1.373124
H 12.154448 -17.697516 0.754412
N 10.482691 -11.085571 1.865507
H 11.272454 -10.602911 1.467164
C 9.515569 -10.529656 2.651589
C 10.194163 -12.392538 1.735632
H 10.781497 -13.079922 1.160076
N 9.099579 -12.698442 2.397033
C 8.660205 -11.540902 2.975327
H 7.759460 -11.489652 3.556187
C 3.573851 -10.847838 4.893523
H 2.925862 -11.389182 5.576063
H 4.222474 -10.169536 5.438898
C 4.419706 -11.831710 4.081608
O 3.813766 -12.878759 3.718010
O 5.597426 -11.554730 3.835038
Ni 8.312301 -14.671422 2.518490
Ni 4.598806 -14.240565 2.287908
O 6.506602 -14.102690 3.233480
H 6.438837 -13.169819 3.498887
O 4.485156 -15.638068 4.079620
H 3.937991 -15.053551 4.618992
H 5.399441 -15.286043 4.151379
C 7.063970 -17.345621 3.497835
O 8.019685 -16.558785 3.411056
N 7.028308 -18.225393 4.531719
H 7.733773 -18.161885 5.242987
N 6.100632 -17.444306 2.576485
H 6.243581 -18.831275 4.683549
H 6.000916 -16.662528 1.915962
H 5.254068 -17.939285 2.797828
H 2.955801 -10.279043 4.202282
H 3.853183 -9.527738 0.236860
H -0.560203 -15.082267 1.698225
H 7.104931 -13.466687 -2.681975
H 10.065908 -15.989833 0.181834
H 9.514112 -9.493830 2.918467

[1]TS1A (249*i* cm^{-1})

N 5.776670 -10.483057 0.516396
H 6.551412 -9.852798 0.386051
C 4.454190 -10.147503 0.474997
C 5.850423 -11.799812 0.782493
H 6.776675 -12.350220 0.873084
N 4.651647 -12.315912 0.908180
C 3.766325 -11.298619 0.719548
H 2.708914 -11.458232 0.773322
N 0.546749 -15.215855 0.134936

H -0.029400 -15.766392 -0.481527
C 0.097953 -14.304723 1.046615
C 1.889122 -15.247205 0.221328
H 2.526523 -15.870772 -0.372115
N 2.314794 -14.406455 1.132421
C 1.210224 -13.809480 1.659727
H 1.296356 -13.083618 2.442655
C 6.954662 -15.328416 -2.991599
H 7.866008 -15.128540 -2.430990
H 6.810561 -14.538640 -3.733606
N 5.863985 -15.366430 -2.052826
H 4.937586 -15.549354 -2.402052
C 5.970805 -15.063097 -0.720676
O 4.911322 -15.152100 -0.059352
O 7.125877 -14.768058 -0.305735
N 9.857191 -14.827283 0.300420
C 10.328030 -14.380926 -0.897855
C 10.333435 -16.042097 0.458756
H 10.147045 -16.659165 1.316238
N 11.099580 -16.393133 -0.589034
H 11.578082 -17.271097 -0.714095
C 11.105766 -15.347443 -1.464175
H 11.643540 -15.370733 -2.388701
N 10.763942 -11.858654 3.650702
H 11.695586 -11.618271 3.949785
C 9.603831 -11.479927 4.259349
C 10.435816 -12.625794 2.598150
H 11.153766 -13.063576 1.933523
N 9.129140 -12.750604 2.497229
C 8.593847 -12.039110 3.535165
H 7.536624 -11.982806 3.711961
C 3.608056 -11.588964 5.239681
H 4.197819 -11.471189 6.143725
H 3.457194 -10.611019 4.787035
C 4.365027 -12.465217 4.240311
O 3.642340 -13.143505 3.461941
O 5.601176 -12.456095 4.254058
Ni 8.152534 -14.219294 1.380906
Ni 4.310717 -14.201188 1.739753
O 6.308048 -14.340893 2.460796
H 6.251353 -13.730460 3.231831
O 3.901241 -15.908568 3.182906
H 3.197473 -15.479876 3.683877
H 4.710706 -15.818712 3.723651
C 7.114114 -16.017689 2.961429
O 8.316726 -15.639666 2.842822
N 6.546491 -16.155647 4.246016
H 7.120378 -15.712379 4.951696
N 6.574083 -16.880035 2.037379
H 6.333262 -17.115863 4.479432
H 6.916942 -16.690670 1.103195
H 5.564670 -16.953531 2.052140
H 2.640079 -12.025508 5.466063
H 4.118544 -9.150223 0.281625
H -0.941111 -14.088817 1.181490
H 7.049729 -16.285602 -3.508937
H 10.084031 -13.407116 -1.272011
H 9.584384 -10.862040 5.132064

[1]Int1A

N 5.669048 -10.445459 0.501371
H 6.435787 -9.816886 0.323274
C 4.342462 -10.135571 0.419375
C 5.760764 -11.737745 0.864320
H 6.697480 -12.261341 0.997568
N 4.568181 -12.262998 1.012887
C 3.669503 -11.276937 0.739888
H 2.614126 -11.448718 0.793982
N 0.453896 -15.157398 0.195132
H -0.129061 -15.706271 -0.416615
C 0.013340 -14.284166 1.146702
C 1.798050 -15.158164 0.235813
H 2.431064 -15.747975 -0.396009
N 2.232220 -14.331301 1.156734
C 1.132314 -13.777278 1.737983
H 1.225796 -13.070960 2.537877
C 7.014780 -15.378196 -2.924933
H 7.902887 -15.103003 -2.358927
H 6.870837 -14.660536 -3.737222
N 5.897793 -15.373143 -2.017510
H 4.995914 -15.652786 -2.367059
C 5.943240 -14.912055 -0.728432
O 4.864012 -14.988959 -0.093895
O 7.057074 -14.479641 -0.324205
N 9.850947 -14.883835 0.319661
C 10.405743 -14.609687 -0.894541

C 10.299929 -16.067466 0.679769
H 10.040015 -16.553665 1.601090
N 11.127547 -16.562327 -0.255525
H 11.600734 -17.451609 -0.224440
C 11.206067 -15.649641 -1.265660
H 11.803798 -15.803499 -2.139448
N 10.808123 -11.748224 3.571460
H 11.742118 -11.481998 3.839912
C 9.670250 -11.502969 4.281328
C 10.457492 -12.429817 2.468730
H 11.154539 -12.754010 1.721333
N 9.155780 -12.624710 2.431083
C 8.649083 -12.051257 3.564849
H 7.603839 -12.076913 3.807088
C 3.800381 -11.862799 5.512120
H 4.327816 -12.032555 6.446377
H 3.885151 -10.809309 5.253337
C 4.447252 -12.683129 4.401735
O 3.666700 -13.198630 3.566070
O 5.688868 -12.786281 4.393613
Ni 8.199871 -14.083855 1.316047
Ni 4.205482 -14.186210 1.757782
O 6.290464 -14.367240 2.453844
H 6.168691 -13.769727 3.286308
O 3.811008 -15.967459 3.058355
H 3.162259 -15.630423 3.685734
H 4.654314 -16.065216 3.563953
C 6.939561 -15.655306 2.840999
O 8.242666 -15.407757 2.849065
N 6.364159 -16.007095 4.134964
H 6.704093 -15.356321 4.836942
N 6.581134 -16.693754 1.924326
H 6.667999 -16.940427 4.385331
H 7.092092 -16.568194 1.056998
H 5.586051 -16.694021 1.726884
H 2.751185 -12.122785 5.610793
H 3.993568 -9.161032 0.149137
H -1.025616 -14.098710 1.321774
H 7.153062 -16.372648 -3.353980
H 10.199676 -13.696860 -1.416408
H 9.672112 -10.974839 5.211363

^{1}TS2A (-504*i* cm^{-1})

N 6.404648 -10.732524 0.594078
H 7.213736 -10.294672 0.184254
C 5.498217 -10.133646 1.419190
C 6.028266 -12.018566 0.443910
H 6.537764 -12.723065 -0.192402
N 4.932010 -12.262340 1.124310
C 4.587445 -11.097214 1.738795
H 3.717471 -11.033463 2.360047
N 0.627624 -13.567597 -0.183845
H 0.055303 -13.467329 -1.007017
C 0.183668 -13.540006 1.106612
C 1.960681 -13.752580 -0.147875
H 2.585911 -13.822897 -1.015209
N 2.382483 -13.843839 1.090193
C 1.287685 -13.714204 1.887360
H 1.376397 -13.757819 2.952946
C 6.993169 -16.976898 -2.282783
H 7.895260 -16.554961 -1.842653
H 6.895307 -16.611989 -3.309620
N 5.881333 -16.574785 -1.458633
H 4.962551 -16.893981 -1.724452
C 5.922569 -15.484112 -0.615487
O 4.820214 -15.162420 -0.109308
O 7.046962 -14.945041 -0.448962
N 9.790024 -14.246542 0.050101
C 10.145050 -13.793552 -1.185286
C 10.465270 -15.358486 0.245152
H 10.398709 -15.954102 1.135508
N 11.246282 -15.634149 -0.814451
H 11.858499 -16.427747 -0.918097
C 11.054123 -14.647479 -1.735949
H 11.560631 -14.630671 -2.678082
N 9.909309 -11.686387 4.019114
H 10.727158 -11.396452 4.531355
C 8.617048 -11.576141 4.443064
C 9.887996 -12.272706 2.808713
H 10.766359 -12.485095 2.232020
N 8.654599 -12.533381 2.434783
C 7.846975 -12.103937 3.450867
H 6.783204 -12.227211 3.410551
C 3.472107 -12.315885 5.680053
H 2.717784 -11.774682 5.118030

H 2.988106 -12.942077 6.425847
C 4.284743 -13.214570 4.752953
O 3.849003 -13.394953 3.609243
O 5.338142 -13.692325 5.258593
Ni 8.026422 -14.041640 1.141342
Ni 4.353600 -14.191292 1.737112
O 6.350037 -14.491263 2.311733
H 6.040553 -14.601514 4.662871
O 3.713635 -16.186886 2.423216
H 3.398236 -16.592950 1.609214
H 4.624970 -16.540034 2.569384
C 6.981523 -15.565041 2.806934
O 8.280956 -15.511455 2.514576
N 6.796040 -15.539012 4.344486
H 7.703551 -15.370644 4.771312
N 6.345649 -16.840736 2.406549
H 6.392844 -16.403944 4.693423
H 6.368531 -16.867230 1.390776
H 6.930937 -17.606651 2.728037
H 4.134768 -11.625936 6.194413
H 5.567925 -9.104018 1.701630
H -0.847705 -13.405275 1.356624
H 7.066655 -18.065115 -2.298531
H 9.736223 -12.893327 -1.597318
H 8.353027 -11.148068 5.386878

^{1}PA

N 6.035443 -10.510432 0.862232
H 6.836803 -9.901907 0.822355
C 4.735403 -10.114816 0.971715
C 6.053691 -11.851967 0.909779
H 6.957549 -12.450171 0.841782
N 4.838542 -12.332491 1.028332
C 4.000746 -11.257671 1.071042
H 2.940788 -11.370459 1.176170
N 0.624981 -14.362605 0.221392
H -0.016092 -14.542858 -0.534834
C 0.279130 -14.021511 1.496641
C 1.968340 -14.415220 0.167092
H 2.534066 -14.658128 -0.709718
N 2.489741 -14.126707 1.335600
C 1.451147 -13.878965 2.178305
H 1.622665 -13.616315 3.201484
C 7.032577 -16.121250 -2.832905
H 7.909840 -15.642610 -2.401975
H 6.771661 -15.614196 -3.765847
N 5.969992 -16.025011 -1.866468
H 5.075647 -16.423815 -2.102906
C 6.028785 -15.257336 -0.731868
O 4.983748 -15.238975 -0.038076
O 7.113341 -14.658594 -0.509070
N 9.906747 -14.474513 -0.091221
C 10.403931 -13.797076 -1.163949
C 10.357786 -15.707076 -0.184831
H 10.140784 -16.486871 0.520005
N 11.132754 -15.846021 -1.274250
H 11.599086 -16.687532 -1.574190
C 11.172348 -14.640502 -1.910489
H 11.724810 -14.480876 -2.812463
N 10.607707 -12.409937 4.066090
H 11.474726 -12.324952 4.572462
C 9.391931 -11.941334 4.472176
C 10.431588 -13.014193 2.879854
H 11.219718 -13.477755 2.319851
N 9.171225 -12.947598 2.504796
C 8.503940 -12.280380 3.495593
H 7.443591 -12.096429 3.454757
C 4.099219 -12.036241 5.619199
H 3.259753 -12.676162 5.872926
H 4.900280 -12.165458 6.343215
C 4.639897 -12.371915 4.219433
O 4.113609 -13.403006 3.696702
O 5.504742 -11.657889 3.730053
Ni 8.228309 -14.138245 1.097645
Ni 4.525626 -14.219392 1.828265
O 6.554711 -14.585695 2.420465
H 4.347098 -14.957782 4.738279
O 4.025765 -16.363615 2.299305
H 4.124459 -16.622451 1.373412
H 4.814922 -16.685465 2.762841
C 7.182431 -15.640267 2.756901
O 8.316481 -15.868155 2.245862
N 4.664402 -15.753713 5.296292
H 4.855959 -15.425167 6.234360
N 6.639565 -16.531227 3.617585
H 3.899737 -16.415619 5.342683

H 7.267344 -17.260410 3.918716
H 5.924482 -16.205616 4.318881
H 3.791666 -10.994212 5.631518
H 4.443674 -9.085594 0.973772
H -0.738832 -13.912793 1.807248
H 7.254766 -17.168278 -3.048404
H 10.184898 -12.761860 -1.329056
H 9.254643 -11.415163 5.393113

[1]**TB** (-22*i* cm^{-1})

N 5.469263 -10.595753 0.649117
H 6.120726 -9.853347 0.453256
C 4.115413 -10.464304 0.746552
C 5.774419 -11.876011 0.918930
H 6.770218 -12.278318 0.918375
N 4.685891 -12.565837 1.173594
C 3.640374 -11.699613 1.071074
H 2.630048 -12.014892 1.235869
N 1.377817 -16.207524 0.663329
H 0.925948 -16.821640 0.004731
C 0.747631 -15.381819 1.547718
C 2.702948 -16.047792 0.833409
H 3.453362 -16.564569 0.269681
N 2.950039 -15.169419 1.776784
C 1.737708 -14.741763 2.231953
H 1.667400 -14.010161 3.011580
C 7.799825 -14.725841 -2.358651
H 8.123581 -15.670616 -2.805075
H 8.612146 -14.317376 -1.761354
N 6.667953 -14.908575 -1.486851
H 5.795031 -15.202475 -1.894587
C 6.747637 -14.958350 -0.121824
O 5.683015 -15.267659 0.471873
O 7.867131 -14.692538 0.391092
N 10.310443 -15.569562 2.049329
C 10.753680 -16.007473 0.835704
C 10.935545 -16.290720 2.951447
H 10.811963 -16.197583 4.011849
N 11.769540 -17.170405 2.369737
H 12.352189 -17.845397 2.838878
C 11.663407 -17.005406 1.020192
H 12.224296 -17.590935 0.322339
N 10.646703 -11.053102 1.864455
H 11.457177 -10.562077 1.521718
C 9.615011 -10.504450 2.568205
C 10.388721 -12.365636 1.728017
H 11.030593 -13.050493 1.210604
N 9.251274 -12.682138 2.306470
C 8.752031 -11.525494 2.836747
H 7.818444 -11.484377 3.364576
C 3.671196 -11.498901 5.403581
H 3.300189 -12.232122 6.114151
H 4.272536 -10.750535 5.909978
C 4.505702 -12.186504 4.325061
O 4.010011 -13.272016 3.883625
O 5.560798 -11.677429 3.948669
Ni 8.441795 -14.637565 2.349735
Ni 4.828144 -14.331553 2.219034
O 6.651005 -14.038311 3.072891
H 6.565549 -13.133179 3.410145
O 3.740971 -15.885336 4.725220
H 2.784692 -15.939987 4.770058
H 3.941573 -14.945454 4.552170
C 6.777473 -16.645161 3.695855
O 7.934209 -16.445919 3.279834
N 6.526917 -16.818586 5.014241
H 7.249074 -16.549690 5.660541
N 5.732630 -16.842555 2.869627
H 5.570349 -16.730232 5.324679
H 5.856595 -16.593667 1.892780
H 4.802440 -16.865412 3.267936
H 2.821200 -11.015817 4.926347
H 3.612348 -9.534203 0.584175
H -0.317889 -15.317957 1.618119
H 7.547137 -14.026091 -3.156189
H 10.380577 -15.584836 -0.074997
H 9.577935 -9.466073 2.822534

[1]**PB**

N 5.090029 -10.544111 0.887318
H 5.662970 -9.744971 0.670180
C 3.745111 -10.522988 1.110501
C 5.511274 -11.808081 1.066202
H 6.530082 -12.130162 0.959198
N 4.505331 -12.590469 1.382532
C 3.394573 -11.804206 1.414909

H 2.429650 -12.202010 1.656050
N 1.359227 -16.154590 0.606803
H 0.896726 -16.650598 -0.138203
C 0.747962 -15.569666 1.677426
C 2.682283 -15.941681 0.731867
H 3.421251 -16.278609 0.033283
N 2.945132 -15.258150 1.820010
C 1.747046 -15.016075 2.421174
H 1.689764 -14.465149 3.337060
C 7.815370 -14.758954 -2.322872
H 8.097899 -15.719557 -2.764449
H 8.640686 -14.388541 -1.718472
N 6.667901 -14.882460 -1.461083
H 5.792365 -15.158020 -1.875946
C 6.736271 -14.941330 -0.091206
O 5.662733 -15.218248 0.492394
O 7.865113 -14.714559 0.422144
N 10.223081 -15.635817 2.209549
C 10.612659 -16.260759 1.061449
C 10.876089 -16.220675 3.186817
H 10.800075 -15.962903 4.224464
N 11.677699 -17.192473 2.715562
H 12.275365 -17.794692 3.259008
C 11.519286 -17.233191 1.361663
H 12.046208 -17.927289 0.741153
N 10.542329 -11.107932 1.428796
H 11.256907 -10.633645 0.899647
C 9.683065 -10.526132 2.314034
C 10.267676 -12.424366 1.404115
H 10.785461 -13.135833 0.792225
N 9.282462 -12.710719 2.225014
C 8.903055 -11.533055 2.801320
H 8.107046 -11.476900 3.517595
C 3.652209 -12.193951 5.921239
H 2.598888 -12.453344 5.859062
H 4.106211 -12.752742 6.737286
C 4.368612 -12.560067 4.619442
O 3.993183 -13.663284 4.103190
O 5.234445 -11.816461 4.167827
Ni 8.393953 -14.606129 2.388436
Ni 4.834327 -14.414873 2.304123
O 6.664953 -13.878212 3.090374
H 6.516804 -12.965082 3.373140
O 3.636462 -16.012610 5.379071
H 3.739266 -15.155908 4.905090
H 2.974017 -15.868895 6.056733
C 6.600017 -16.441459 3.869693
O 7.735586 -16.358132 3.385378
N 6.382108 -16.533746 5.192714
H 7.135419 -16.285704 5.811218
N 5.472436 -16.526467 3.087422
H 5.426087 -16.422711 5.525819
H 5.640039 -16.748513 2.111244
H 4.630978 -16.880447 3.527094
H 3.768006 -11.132015 6.113360
H 3.162665 -9.628365 1.040611
H -0.311943 -15.595330 1.819999
H 7.604843 -14.050220 -3.124971
H 10.206991 -15.974321 0.112253
H 9.695870 -9.477816 2.526857

^{2}R

N 4.868788 -10.191426 1.042163
H 5.387739 -9.342121 0.888236
C 3.543804 -10.268450 1.352932
C 5.358024 -11.443409 1.040663
H 6.379827 -11.694397 0.828835
N 4.414168 -12.310872 1.324376
C 3.273986 -11.593287 1.522691
H 2.347544 -12.065658 1.780172
N 1.292019 -15.519532 0.151559
H 0.777060 -15.770934 -0.677101
C 0.760575 -15.323767 1.393513
C 2.618398 -15.309969 0.242566
H 3.307520 -15.391201 -0.573366
N 2.958184 -14.997205 1.469969
C 1.808927 -14.999669 2.202334
H 1.820630 -14.763577 3.246968
C 8.102120 -14.679771 -2.445164
H 8.218315 -15.669865 -2.897177
H 8.936241 -14.498508 -1.770586
N 6.889763 -14.576657 -1.674132
H 6.014903 -14.668692 -2.165891
C 6.833621 -14.756815 -0.309656
O 5.683034 -14.844033 0.173022
O 7.942903 -14.824408 0.285757

N 10.188168 -15.732388 2.231365
C 10.558083 -16.503003 1.168676
C 10.813496 -16.221010 3.277381
H 10.740873 -15.840661 4.276938
N 11.578855 -17.271247 2.931324
H 12.150440 -17.826890 3.547672
C 11.425233 -17.466251 1.590531
H 11.927855 -18.248256 1.061078
N 10.673221 -11.270438 0.968426
H 11.343408 -10.853271 0.341962
C 9.946257 -10.611583 1.915635
C 10.337331 -12.571131 1.032251
H 10.751319 -13.332487 0.401616
N 9.438011 -12.774706 1.968260
C 9.180451 -11.558401 2.529456
H 8.475942 -11.439018 3.328594
C 3.681952 -12.499715 5.880367
H 3.226921 -13.417951 6.239968
H 4.405404 -12.125512 6.599262
C 4.372641 -12.725241 4.531798
O 4.082595 -13.824962 3.963715
O 5.137909 -11.869722 4.093663
Ni 8.435108 -14.583578 2.246932
Ni 4.851210 -14.203918 2.027476
O 6.712507 -13.690120 2.788494
H 6.603013 -12.790835 3.121479
C 6.414768 -16.116063 3.648801
O 7.611359 -16.204446 3.364361
N 5.960194 -15.858603 4.898700
H 6.649284 -15.488828 5.536467
N 5.428606 -16.357163 2.718003
H 5.057600 -15.394473 4.937075
H 5.756286 -16.710461 1.826867
H 4.530214 -16.685364 3.046229
H 2.911874 -11.743811 5.745031
H 2.918118 -9.404113 1.429680
H -0.284562 -15.429216 1.595780
H 8.101623 -13.929011 -3.236258
H 10.167802 -16.315858 0.188705
H 10.030285 -9.557536 2.078224

²TS1A (-287*i* cm⁻¹)

N 5.252270 -10.028884 0.238999
H 5.941807 -9.326252 0.024959
C 3.940170 -9.797778 0.531032
C 5.459052 -11.353945 0.313483
H 6.403416 -11.837567 0.133062
N 4.345954 -11.976305 0.631770
C 3.386935 -11.019605 0.773125
H 2.379507 -11.268970 1.038892
N 0.665823 -15.332792 -0.117433
H 0.091361 -15.771334 -0.819580
C 0.230810 -14.845040 1.080581
C 1.991565 -15.121221 -0.183849
H 2.614399 -15.397976 -1.010618
N 2.422319 -14.528152 0.905804
C 1.333735 -14.346247 1.706278
H 1.428673 -13.878983 2.665298
C 7.271203 -15.425930 -3.205931
H 8.096022 -14.946877 -2.681660
H 7.065759 -14.874265 -4.126819
N 6.132386 -15.414759 -2.326078
H 5.273629 -15.827982 -2.651386
C 6.098269 -14.773430 -1.117227
O 5.005386 -14.850407 -0.498601
O 7.144299 -14.176038 -0.755404
N 9.829570 -14.809447 -0.046386
C 10.614470 -14.315917 -1.045567
C 9.919652 -16.120079 -0.112131
H 9.412803 -16.791915 0.553382
N 10.738853 -16.488813 -1.112084
H 10.978388 -17.432281 -1.372777
C 11.189769 -15.353333 -1.718310
H 11.861010 -15.370074 -2.551149
N 10.876776 -12.344372 3.767903
H 11.784574 -12.310497 4.203904
C 9.759696 -11.686500 4.190782
C 10.541057 -13.046822 2.672479
H 11.226002 -13.664039 2.125453
N 9.272572 -12.869588 2.371705
C 8.768025 -12.019966 3.317090
H 7.741263 -11.709274 3.328887
C 3.554568 -12.052371 5.176007
H 4.242595 -11.796182 5.975844
H 2.972147 -11.170253 4.918068
C 4.342370 -12.481044 3.940088

O 3.772450 -13.339464 3.209571
O 5.452329 -11.976189 3.731603
Ni 8.200278 -14.000049 0.972936
Ni 4.372072 -13.908183 1.283205
O 6.373756 -13.731595 2.003117
H 6.246680 -13.068695 2.730206
C 6.694469 -15.362251 2.704843
O 7.788398 -15.633272 2.088523
N 6.767312 -15.084031 4.065559
H 7.698058 -15.086989 4.444844
N 5.546805 -16.075034 2.349276
H 6.049893 -15.464014 4.657116
H 5.465205 -16.235456 1.351922
H 4.687229 -15.778702 2.801827
H 2.879993 -12.844188 5.486794
H 3.516249 -8.815636 0.544778
H -0.794131 -14.890957 1.383320
H 7.546206 -16.451539 -3.462265
H 10.717574 -13.263049 -1.215356
H 9.754072 -11.051490 5.051342

[2]Int1A

N 5.388758 -10.179519 0.523791
H 6.077449 -9.552630 0.140306
C 4.129224 -9.836898 0.923223
C 5.536879 -11.501054 0.728138
H 6.427573 -12.061137 0.503131
N 4.437394 -12.012002 1.233722
C 3.548310 -10.988871 1.362960
H 2.567877 -11.147860 1.764158
N 0.697474 -15.069914 0.258480
H 0.119488 -15.333037 -0.523918
C 0.276352 -14.920855 1.547877
C 2.014688 -14.800606 0.228320
H 2.627017 -14.835383 -0.650250
N 2.450686 -14.492090 1.427374
C 1.378713 -14.561239 2.264490
H 1.477587 -14.340092 3.307380
C 7.586635 -15.701997 -2.293386
H 8.390692 -15.238368 -1.725218
H 7.505953 -15.211037 -3.267120
N 6.376413 -15.557390 -1.529551
H 5.533619 -15.979139 -1.885988
C 6.240041 -14.744644 -0.435100
O 5.085145 -14.683735 0.046175
O 7.265890 -14.101491 -0.062599
N 9.823993 -15.423625 0.892947
C 10.658915 -15.193410 -0.161453
C 9.966700 -16.692536 1.211367
H 9.426851 -17.181848 1.999208
N 10.864904 -17.284420 0.406968
H 11.154738 -18.249014 0.442894
C 11.315723 -16.346593 -0.473668
H 12.043695 -16.562803 -1.227180
N 10.529198 -10.778150 2.279673
H 11.381545 -10.242758 2.231877
C 9.374274 -10.391979 2.892656
C 10.324730 -12.006184 1.771533
H 11.071863 -12.559527 1.238355
N 9.104425 -12.423442 2.023665
C 8.495536 -11.420775 2.726711
H 7.485779 -11.503643 3.078553
C 3.522399 -11.836204 5.691961
H 2.615713 -12.393601 5.905806
H 4.120515 -11.712839 6.589866
C 4.335662 -12.549497 4.620773
O 3.694044 -13.206052 3.770878
O 5.581987 -12.435405 4.661825
Ni 8.238767 -14.332015 1.678341
Ni 4.379987 -13.974710 1.915714
O 6.441800 -13.928081 2.825156
H 6.188557 -13.295483 3.631293
C 6.425229 -15.348195 3.226103
O 7.438230 -15.922725 2.599791
N 6.484868 -15.515642 4.647987
H 7.428321 -15.383181 4.987758
N 5.098983 -15.847349 2.776655
H 5.853698 -14.888087 5.130211
H 5.249427 -16.503256 2.012772
H 4.625835 -16.320649 3.541767
H 3.249256 -10.853648 5.312473
H 3.758065 -8.835230 0.864988
H -0.739424 -15.078938 1.843900
H 7.810263 -16.759169 -2.447768
H 10.724838 -14.232559 -0.631027
H 9.271130 -9.450067 3.388946

^{2}TS2A (691*i* cm^{-1})

N 5.536922 -10.053006 0.151138
H 6.256160 -9.414805 -0.149694
C 4.222467 -9.742012 0.346771
C 5.690902 -11.360874 0.422253
H 6.623919 -11.898915 0.358641
N 4.545342 -11.894952 0.778114
C 3.617031 -10.899258 0.737710
H 2.591336 -11.077224 0.990288
N 0.610788 -14.878181 -0.087747
H -0.006735 -15.113411 -0.848376
C 0.256371 -14.769647 1.225409
C 1.926986 -14.617053 -0.174433
H 2.492548 -14.628592 -1.084423
N 2.428506 -14.351796 1.009524
C 1.396554 -14.441409 1.895814
H 1.545315 -14.258554 2.940270
C 7.151793 -15.734275 -3.053030
H 7.979710 -15.166171 -2.632786
H 6.949902 -15.378008 -4.065930
N 6.011765 -15.531012 -2.198692
H 5.152507 -16.006807 -2.420914
C 6.020514 -14.742277 -1.080738
O 4.928830 -14.701843 -0.450459
O 7.080158 -14.124173 -0.815826
N 9.848218 -14.610890 -0.143003
C 10.577352 -14.122639 -1.185709
C 10.013629 -15.915817 -0.150363
H 9.559453 -16.581609 0.558672
N 10.827504 -16.285114 -1.155015
H 11.114675 -17.224764 -1.378233
C 11.195671 -15.155877 -1.825731
H 11.845408 -15.174397 -2.675434
N 10.677493 -12.009933 3.707232
H 11.573494 -11.835616 4.134179
C 9.464682 -11.641045 4.211134
C 10.454938 -12.652861 2.546982
H 11.232014 -13.056872 1.928828
N 9.168549 -12.709094 2.282154
C 8.534385 -12.079719 3.316886
H 7.464906 -12.008200 3.352491
C 3.516988 -11.688643 5.009080
H 2.968070 -12.125415 5.841057
H 4.183741 -10.928440 5.404673
C 4.322408 -12.784747 4.337034
O 3.851769 -13.338423 3.332645
O 5.419297 -13.080793 4.901585
Ni 8.169892 -13.905333 0.886718
Ni 4.438610 -13.856650 1.389359
O 6.413988 -13.859726 2.053666
H 6.067558 -14.024654 4.426441
C 6.678102 -15.124070 2.501683
O 7.810040 -15.565956 1.969423
N 6.801450 -15.018448 4.030900
H 7.773284 -14.817138 4.250433
N 5.565144 -16.050983 2.322791
H 6.530094 -15.901473 4.454105
H 5.522698 -16.378325 1.365456
H 4.675964 -15.649668 2.630713
H 2.812937 -11.256225 4.305116
H 3.834397 -8.756233 0.197362
H -0.744063 -14.931396 1.567793
H 7.419561 -16.792852 -3.095794
H 10.613370 -13.075306 -1.408234
H 9.359750 -11.112710 5.135138

^{2}Int3A

N 5.642261 -10.065003 0.226445
H 6.375937 -9.440822 -0.068754
C 4.354231 -9.709526 0.505271
C 5.747228 -11.392833 0.410809
H 6.651193 -11.965182 0.269929
N 4.595445 -11.896726 0.789294
C 3.713825 -10.860891 0.855398
H 2.694518 -11.009973 1.148904
N 0.589614 -14.716516 -0.149144
H -0.021052 -14.954481 -0.914475
C 0.206462 -14.505584 1.143594
C 1.922443 -14.549498 -0.208626
H 2.511172 -14.652625 -1.097947
N 2.406713 -14.247495 0.973902
C 1.346764 -14.214201 1.830391
H 1.482652 -13.980466 2.866430
C 7.139707 -15.972906 -2.953224
H 7.968853 -15.381143 -2.569647
H 6.933627 -15.675705 -3.984312

N 6.003969 -15.721804 -2.106417
H 5.142117 -16.204984 -2.301680
C 6.008928 -14.852847 -1.048734
O 4.919995 -14.765641 -0.423699
O 7.071153 -14.215010 -0.831679
N 9.867453 -14.596097 -0.178691
C 10.601189 -14.074487 -1.202036
C 10.067159 -15.896420 -0.200870
H 9.619178 -16.584418 0.490635
N 10.907226 -16.229899 -1.196026
H 11.222941 -17.158518 -1.427045
C 11.257003 -15.082483 -1.844895
H 11.921074 -15.072016 -2.683552
N 10.604889 -11.802996 3.591068
H 11.492426 -11.530658 3.983109
C 9.377587 -11.528527 4.119760
C 10.410750 -12.506101 2.461267
H 11.204726 -12.860456 1.834045
N 9.127892 -12.690534 2.239618
C 8.466429 -12.083822 3.271863
H 7.395657 -12.106566 3.339646
C 3.382596 -11.891537 5.150252
H 2.850914 -12.478714 5.896203
H 3.981570 -11.145060 5.663063
C 4.293506 -12.830070 4.362695
O 3.815477 -13.296943 3.300627
O 5.410704 -13.086839 4.849051
Ni 8.177307 -13.934120 0.846824
Ni 4.424097 -13.842243 1.395503
O 6.417503 -13.931736 2.008870
H 6.239345 -14.207812 4.349833
C 6.693584 -15.184087 2.413067
O 7.819265 -15.625380 1.906814
N 6.875631 -15.053915 4.023902
H 7.854572 -14.849999 4.210614
N 5.588736 -16.117689 2.346531
H 6.599498 -15.926847 4.466956
H 5.551557 -16.552688 1.433448
H 4.701779 -15.650971 2.563656
H 2.663592 -11.420567 4.487302
H 4.006336 -8.700240 0.434754
H -0.812081 -14.579435 1.462011
H 7.407276 -17.032214 -2.935920
H 10.613083 -13.023800 -1.410997
H 9.249193 -10.977928 5.027586

^{2}TS4A (-181*i* cm^{-1})

N 5.870987 -10.123131 0.516808
H 6.646140 -9.536472 0.252737
C 4.667372 -9.690966 0.993554
C 5.830364 -11.466214 0.466297
H 6.644358 -12.092352 0.131733
N 4.665963 -11.907514 0.884121
C 3.926958 -10.812936 1.218578
H 2.928021 -10.905067 1.594263
N 0.576585 -14.363927 -0.381822
H -0.009097 -14.535464 -1.183651
C 0.143035 -14.163851 0.896697
C 1.919031 -14.290265 -0.370653
H 2.542405 -14.412148 -1.233767
N 2.360965 -14.056943 0.843865
C 1.263802 -13.973671 1.648128
H 1.365284 -13.784980 2.697271
C 7.107767 -16.446354 -2.692191
H 7.931651 -15.802216 -2.390092
H 6.901762 -16.291872 -3.754110
N 5.968659 -16.092173 -1.888245
H 5.111285 -16.606043 -2.013374
C 5.963554 -15.089179 -0.957831
O 4.875492 -14.923060 -0.350826
O 7.020052 -14.413144 -0.831861
N 9.852280 -14.548345 -0.227274
C 10.514691 -14.025383 -1.297829
C 10.179586 -15.821957 -0.171054
H 9.814219 -16.505656 0.571362
N 11.032534 -16.135632 -1.161293
H 11.434997 -17.042068 -1.340125
C 11.256667 -15.004614 -1.889291
H 11.902967 -14.984130 -2.741508
N 10.431650 -11.478143 3.355766
H 11.299700 -11.112858 3.714408
C 9.191372 -11.260936 3.882115
C 10.281381 -12.276564 2.284613
H 11.094737 -12.614327 1.672979
N 9.013602 -12.574344 2.097545
C 8.315865 -11.945196 3.092970

H 7.250967 -12.049588 3.193220
C 3.242616 -12.439857 5.365902
H 2.643336 -13.292813 5.672835
H 3.775708 -12.031619 6.218644
C 4.244221 -12.883093 4.293439
O 3.716112 -13.438160 3.289311
O 5.446615 -12.686067 4.481686
Ni 8.139607 -13.951923 0.794659
Ni 4.376822 -13.859479 1.387098
O 6.377950 -14.040488 1.960159
H 6.498000 -14.022692 4.316157
C 6.730394 -15.241084 2.345347
O 7.869251 -15.645557 1.904749
N 7.063050 -14.875597 4.144973
H 8.050046 -14.689596 4.269242
N 5.716433 -16.194622 2.545717
H 6.759267 -15.645180 4.728072
H 5.832907 -17.020863 1.980273
H 4.784840 -15.782707 2.475243
H 2.582607 -11.685731 4.945125
H 4.439565 -8.654702 1.130685
H -0.893306 -14.174547 1.161685
H 7.382927 -17.491707 -2.532589
H 10.418964 -12.994711 -1.573765
H 9.029289 -10.656982 4.749634

^{2}PA

N 6.099745 -10.601551 -0.801715
H 6.534325 -10.406945 -1.689093
C 5.586843 -9.676531 0.062764
C 5.884431 -11.818930 -0.268586
H 6.201202 -12.740118 -0.717074
N 5.268305 -11.714752 0.883171
C 5.074501 -10.384044 1.108044
H 4.602233 -10.038077 2.005487
N 0.608523 -13.789649 1.320231
H -0.169345 -14.424908 1.244856
C 0.530247 -12.427906 1.325390
C 1.904616 -14.122169 1.451725
H 2.281132 -15.125304 1.479500
N 2.652060 -13.046069 1.533611
C 1.810430 -11.978890 1.458791
H 2.172196 -10.972465 1.508167
C 7.037645 -17.248131 -0.716727
H 7.915528 -16.624644 -0.549806
H 6.898817 -17.394106 -1.791634
N 5.912621 -16.572183 -0.129246
H 5.041733 -17.068774 -0.032354
C 5.943290 -15.284242 0.323142
O 4.882570 -14.859096 0.840434
O 7.008486 -14.625314 0.119466
N 9.964471 -14.743668 0.750779
C 10.435870 -14.617566 -0.523294
C 10.598869 -15.766413 1.282496
H 10.441008 -16.130901 2.279014
N 11.464219 -16.298031 0.402373
H 12.070677 -17.087053 0.560747
C 11.373016 -15.580430 -0.753053
H 11.961494 -15.802622 -1.618450
N 9.479773 -9.912054 1.446915
H 10.090972 -9.150688 1.197190
C 8.461614 -9.857838 2.354510
C 9.534426 -11.171957 0.977870
H 10.248642 -11.505244 0.250985
N 8.606470 -11.919002 1.532967
C 7.925520 -11.109929 2.400503
H 7.098192 -11.478450 2.978678
C 3.528557 -12.614893 5.956442
H 2.744088 -13.297630 6.269861
H 4.413164 -12.794640 6.565004
C 3.882865 -12.862076 4.510868
O 4.340499 -11.931095 3.807141
O 3.724780 -14.010032 4.016674
Ni 8.234764 -13.981811 1.592774
Ni 4.635399 -13.252002 2.166281
O 6.552659 -13.861073 2.891426
H 5.961775 -19.151457 4.062907
C 6.792987 -15.084211 3.223048
O 7.897805 -15.588103 2.853150
N 6.469868 -18.469265 4.612191
H 6.313944 -18.676090 5.591274
N 5.936080 -15.774057 3.964254
H 7.457384 -18.581400 4.419231
H 6.119218 -16.757265 4.197676
H 5.031912 -15.351971 4.147752
H 3.218327 -11.582839 6.091201

H 5.620723 -8.625235 -0.132933
H -0.397908 -11.903437 1.236885
H 7.184117 -18.218972 -0.240997
H 10.064006 -13.863096 -1.185954
H 8.207458 -8.962946 2.881875

UB3LYP

[1]R

N	5.681998	-10.795214	-0.150389
H	6.359927	-10.209991	-0.624249
C	4.328867	-10.550350	-0.043798
C	5.954916	-11.949568	0.505554
H	6.944812	-12.393921	0.581785
N	4.848802	-12.458454	1.017594
C	3.826431	-11.596730	0.688435
H	2.803045	-11.778787	1.004757
N	1.107782	-15.713906	0.689700
H	0.624251	-16.349069	0.065080
C	0.517910	-14.873668	1.611230
C	2.450906	-15.547510	0.769043
H	3.180494	-16.091259	0.174146
N	2.748116	-14.647365	1.687554
C	1.555269	-14.217050	2.226825
H	1.548508	-13.476265	3.022320
C	7.456553	-15.209672	-2.108758
H	8.313559	-15.877859	-1.916966
H	7.827610	-14.172090	-2.088285
N	6.386878	-15.408330	-1.154723
H	5.509698	-15.803315	-1.465817
C	6.504843	-15.188006	0.186230
O	5.503443	-15.486581	0.920326
O	7.608466	-14.703939	0.593988
N	10.036848	-15.475193	1.931629
C	10.624919	-15.375834	0.689431
C	10.763062	-16.331824	2.627347
H	10.548206	-16.660924	3.641044
N	11.802684	-16.782262	1.885081
H	12.498726	-17.454783	2.188055
C	11.733512	-16.185694	0.643756
H	12.451668	-16.393032	-0.144886
N	10.612696	-11.089880	2.735588
H	11.507815	-10.623948	2.636698
C	9.448257	-10.528300	3.215109
C	10.360210	-12.383828	2.434927
H	11.109637	-13.063627	2.037987
N	9.094998	-12.683502	2.691906
C	8.509821	-11.528984	3.182827
H	7.469553	-11.484154	3.504977
C	3.434382	-10.959305	5.116316
H	2.720195	-11.521361	5.737169
H	4.015814	-10.268506	5.740147
C	4.351343	-11.917536	4.368350
O	3.761889	-12.879614	3.752466
O	5.580128	-11.725226	4.385333
NI	8.188475	-14.605919	2.526901
NI	4.665269	-14.089150	2.326736
O	6.475436	-14.011161	3.270371
H	6.381413	-13.156366	3.751354
O	4.544241	-15.509279	4.107687
H	3.982083	-14.858014	4.560784
H	5.442984	-15.082061	4.144883
C	7.097484	-17.315404	3.694239
O	7.970670	-16.435516	3.524606
N	7.143594	-18.093308	4.810367
H	7.772129	-17.805486	5.548759
N	6.146319	-17.587933	2.775101
H	6.390209	-18.723073	5.053103
H	5.954685	-16.853134	2.079237
H	5.318731	-18.085842	3.082790
H	2.842473	-10.380654	4.388288
H	3.855576	-9.676231	-0.482245
H	-0.558850	-14.820281	1.746663
H	7.077528	-15.418154	-3.118938
H	10.192024	-14.753030	-0.088601
H	9.388456	-9.493986	3.542420

[1]TS1A (-313i cm^{-1})

N	5.583807	-10.429424	0.641211
H	6.136467	-9.785432	0.087840
C	4.544318	-10.094889	1.484300
C	5.742271	-11.775429	0.678779
H	6.498825	-12.324530	0.120146
N	4.860900	-12.319356	1.500307
C	4.105625	-11.283561	2.009431
H	3.300427	-11.468971	2.714333
N	0.747615	-13.734637	-0.284278

H	0.231523	-13.635727	-1.151051
C	0.211423	-13.722472	0.987165
C	2.087657	-13.908381	-0.172462
H	2.772215	-13.965468	-1.015803
N	2.431530	-14.004401	1.098121
C	1.276100	-13.892968	1.837725
H	1.306144	-13.941905	2.923132
C	7.038451	-15.052736	-2.905954
H	7.524597	-14.067051	-2.850651
H	6.652151	-15.186306	-3.926455
N	5.937087	-15.131724	-1.970772
H	5.089908	-15.610809	-2.249054
C	6.038567	-14.855407	-0.632126
O	5.017130	-15.098883	0.077450
O	7.144888	-14.355638	-0.231045
N	9.809773	-15.299935	0.605026
C	10.350736	-14.990129	-0.622910
C	10.584325	-16.225959	1.144904
H	10.397201	-16.701717	2.106141
N	11.610848	-16.517621	0.312027
H	12.339667	-17.200042	0.490487
C	11.483336	-15.741985	-0.821142
H	12.184208	-15.797574	-1.649624
N	9.988873	-11.042080	2.340770
H	10.746572	-10.389158	2.174353
C	9.044263	-10.957639	3.342176
C	9.750814	-12.170665	1.633776
H	10.359391	-12.495153	0.792787
N	8.700868	-12.809761	2.126220
C	8.245917	-12.064898	3.198984
H	7.387864	-12.368202	3.799628
C	3.782875	-12.694647	5.890268
H	2.758447	-13.067181	6.022040
H	4.382943	-12.880008	6.791581
C	4.444429	-13.323266	4.677203
O	3.685642	-13.756413	3.756748
O	5.702812	-13.354558	4.638632
NI	8.060723	-14.690888	1.562585
NI	4.344241	-14.350577	1.904616
O	6.314819	-14.925298	2.769109
H	6.171259	-14.288711	3.586414
O	3.640278	-16.298373	2.259633
H	3.503544	-16.641938	1.364462
H	4.445692	-16.797511	2.590820
C	6.951802	-16.234124	3.288903
O	8.178527	-16.221765	2.765124
N	6.932511	-16.254094	4.690761
H	7.833094	-16.313589	5.143678
N	6.073949	-17.287550	2.741591
H	6.130509	-16.625749	5.181363
H	6.269311	-17.348786	1.739231
H	6.377340	-18.176339	3.143988
H	3.737225	-11.602474	5.741158
H	4.215588	-9.069852	1.631572
H	-0.852604	-13.599847	1.169778
H	7.811756	-15.821135	-2.725527
H	9.877777	-14.271378	-1.287207
H	9.024763	-10.142686	4.060402

[1]Int1A

N	5.617086	-10.399791	0.713289
H	6.176912	-9.761240	0.160603
C	4.570868	-10.056468	1.543856
C	5.766038	-11.747218	0.754126
H	6.514543	-12.305339	0.191846
N	4.873161	-12.283303	1.567301
C	4.118599	-11.243041	2.064848
H	3.293434	-11.416469	2.749598
N	0.751218	-13.696538	-0.115525
H	0.215281	-13.536112	-0.960711
C	0.237885	-13.858622	1.154853
C	2.101176	-13.798835	-0.038232
H	2.774922	-13.731086	-0.889172
N	2.474963	-14.012420	1.209353
C	1.327439	-14.055876	1.967438
H	1.370535	-14.228261	3.039169
C	6.971054	-15.068519	-3.079150
H	7.511307	-14.110257	-3.049273
H	6.572723	-15.206478	-4.094663
N	5.873698	-15.063069	-2.137280
H	4.996594	-15.495353	-2.398150
C	5.995495	-14.726737	-0.812295
O	4.956558	-14.868101	-0.098366
O	7.131329	-14.275883	-0.439898
N	9.783547	-15.317131	0.448701
C	10.367047	-15.088744	-0.777153

C	10.518665	-16.228620	1.063951
H	10.289681	-16.632447	2.049873
N	11.561334	-16.590872	0.279554
H	12.268199	-17.278026	0.517319
C	11.485980	-15.876772	-0.898587
H	12.209896	-15.994386	-1.700376
N	10.068676	-11.132149	2.549951
H	10.846115	-10.482979	2.502837
C	9.062172	-11.131112	3.493300
C	9.854419	-12.172495	1.708895
H	10.508064	-12.421760	0.875425
N	8.763748	-12.830163	2.061935
C	8.256078	-12.196080	3.175941
H	7.356745	-12.554379	3.670615
C	3.461354	-12.328161	5.678036
H	2.434469	-12.251832	5.300736
H	3.456236	-12.635350	6.734512
C	4.266878	-13.312017	4.860662
O	3.790547	-13.842425	3.850080
O	5.461838	-13.520509	5.321422
NI	8.052865	-14.628773	1.371396
NI	4.465268	-14.358199	1.874619
O	6.359010	-14.859084	2.432830
H	5.970618	-14.388218	4.924229
O	3.821038	-16.348924	2.120965
H	3.776566	-16.642413	1.198544
H	4.628134	-16.819679	2.495455
C	6.934441	-15.881864	3.174241
O	8.254716	-15.894849	2.913855
N	6.678538	-15.668814	4.639844
H	7.586338	-15.702484	5.107011
N	6.259379	-17.176393	2.822409
H	6.094284	-16.405622	5.039448
H	6.570696	-17.392283	1.871733
H	6.658958	-17.923902	3.396092
H	3.947544	-11.340442	5.635393
H	4.245072	-9.029317	1.683114
H	-0.828122	-13.824740	1.362340
H	7.703356	-15.873166	-2.883607
H	9.934259	-14.392783	-1.491393
H	9.012021	-10.396734	4.292430

[1]**TS2A** (-474i cm^{-1})

N	5.621500	-10.399353	0.695227
H	6.174070	-9.761360	0.134732
C	4.589543	-10.054668	1.543154
C	5.766604	-11.747407	0.731234
H	6.506349	-12.305877	0.158097
N	4.885084	-12.282117	1.557462
C	4.141856	-11.240671	2.069170
H	3.329332	-11.414149	2.768775
N	0.745865	-13.696686	-0.123066
H	0.206765	-13.559229	-0.970227
C	0.239439	-13.795383	1.156608
C	2.094017	-13.824585	-0.051097
H	2.762984	-13.804233	-0.908207
N	2.472755	-13.995095	1.201560
C	1.330830	-13.982282	1.969134
H	1.381836	-14.110473	3.046687
C	6.967629	-15.094587	-3.079828
H	7.507150	-14.135669	-3.056635
H	6.568372	-15.239051	-4.094040
N	5.871085	-15.084895	-2.137041
H	4.991713	-15.512610	-2.397740
C	5.992874	-14.743357	-0.813506
O	4.954851	-14.877983	-0.098271
O	7.131847	-14.295546	-0.443458
N	9.788646	-15.310337	0.450424
C	10.363465	-15.074131	-0.778167
C	10.538080	-16.212698	1.062000
H	10.320430	-16.620955	2.048459
N	11.581399	-16.561482	0.272645
H	12.297973	-17.239871	0.506681
C	11.491424	-15.848153	-0.904901
H	12.213040	-15.956379	-1.710072
N	10.028056	-11.132512	2.579495
H	10.799410	-10.475702	2.538650
C	9.011609	-11.138994	3.512078
C	9.832541	-12.176306	1.738566
H	10.496949	-12.420715	0.912203
N	8.744361	-12.843693	2.081857
C	8.218133	-12.211768	3.189342
H	7.315499	-12.572693	3.677607
C	3.499670	-12.366274	5.687437
H	2.455893	-12.316052	5.353330
H	3.546521	-12.673071	6.742917

C	4.306437	-13.325794	4.835499
O	3.789998	-13.839530	3.824303
O	5.506221	-13.530386	5.244035
NI	8.052117	-14.637573	1.364988
NI	4.454802	-14.348640	1.879324
O	6.355200	-14.888941	2.415723
H	6.059371	-14.515668	4.861488
O	3.812342	-16.351788	2.096007
H	3.811329	-16.625255	1.166099
H	4.600842	-16.824882	2.493522
C	6.931903	-15.896150	3.158578
O	8.250424	-15.913594	2.916279
N	6.670069	-15.651223	4.640351
H	7.579207	-15.662212	5.107706
N	6.253193	-17.195667	2.850866
H	6.088322	-16.380421	5.058903
H	6.550802	-17.432557	1.900751
H	6.661679	-17.931987	3.433050
H	3.954731	-11.364485	5.626998
H	4.269432	-9.026780	1.689864
H	-0.823926	-13.729934	1.369970
H	7.700728	-15.897489	-2.880281
H	9.918192	-14.383404	-1.489727
H	8.945301	-10.403094	4.308622

^{1}PA

N	5.694551	-10.328360	0.648974
H	6.263474	-9.655070	0.149604
C	4.597150	-10.044321	1.435290
C	5.882916	-11.670972	0.656851
H	6.674327	-12.182122	0.111525
N	4.966930	-12.258768	1.407115
C	4.157866	-11.257790	1.899154
H	3.319485	-11.484226	2.551063
N	0.912480	-13.975244	-0.331167
H	0.387730	-13.893384	-1.194267
C	0.380237	-14.083639	0.937618
C	2.263670	-13.999127	-0.221209
H	2.947038	-13.933462	-1.064248
N	2.618748	-14.113834	1.044287
C	1.458859	-14.168089	1.783141
H	1.500516	-14.254899	2.865265
C	6.696176	-14.515902	-3.293922
H	7.319069	-13.637840	-3.076178
H	6.142709	-14.324509	-4.224858
N	5.757722	-14.731325	-2.212451
H	4.894152	-15.221949	-2.411883
C	6.078036	-14.664492	-0.881908
O	5.198233	-15.029761	-0.053282
O	7.246864	-14.217302	-0.593380
N	9.818286	-15.456029	0.109750
C	10.311352	-15.143505	-1.138347
C	10.566616	-16.438372	0.588194
H	10.415638	-16.925842	1.549923
N	11.527259	-16.765100	-0.305420
H	12.222445	-17.493930	-0.183797
C	11.384826	-15.954330	-1.412408
H	12.038008	-16.027486	-2.277643
N	10.286415	-11.552436	2.772945
H	11.106798	-10.960005	2.837240
C	9.172045	-11.494929	3.585105
C	10.105094	-12.552518	1.881592
H	10.833540	-12.826818	1.121353
N	8.931708	-13.133487	2.081422
C	8.329251	-12.486201	3.147483
H	7.344738	-12.768295	3.533969
C	3.371372	-12.865614	5.773646
H	2.883821	-13.780347	6.147935
H	3.896433	-12.358548	6.594005
C	4.333612	-13.216280	4.636344
O	3.774573	-13.794150	3.637736
O	5.539541	-12.947448	4.749483
NI	8.166480	-14.751897	1.112176
NI	4.557839	-14.284681	1.854036
O	6.556105	-14.860652	2.459948
H	5.987175	-14.374438	6.122447
O	4.135477	-16.372536	2.138722
H	4.478835	-16.707620	1.295272
H	4.740694	-16.726061	2.824825
C	7.083830	-15.922180	2.958649
O	8.195840	-16.310259	2.483681
N	5.924027	-15.359050	6.413726
H	6.691161	-15.540418	7.063611
N	6.430379	-16.646088	3.916542
H	5.062026	-15.450399	6.954384
H	6.982527	-17.444345	4.223670

H	6.053753	-16.121619	4.750984
H	2.565953	-12.214236	5.398664
H	4.237116	-9.031796	1.594839
H	-0.690516	-14.093022	1.121570
H	7.367677	-15.378265	-3.462139
H	9.848828	-14.376505	-1.753715
H	9.073305	-10.774325	4.392114

[1]TB (-143*i* cm^{-1})

N	5.552942	-10.665400	-0.209184
H	6.194221	-10.055185	-0.702796
C	4.203590	-10.448014	-0.024480
C	5.880786	-11.834181	0.391612
H	6.879088	-12.269135	0.401340
N	4.809190	-12.376435	0.945176
C	3.755793	-11.524526	0.699417
H	2.753567	-11.736613	1.061991
N	1.285831	-15.990103	0.658851
H	0.828911	-16.653212	0.042797
C	0.670481	-15.216887	1.621420
C	2.611865	-15.717679	0.682766
H	3.356216	-16.182585	0.041531
N	2.877101	-14.812234	1.609250
C	1.676509	-14.488681	2.206275
H	1.644149	-13.759915	3.011456
C	7.672945	-15.137987	-2.248775
H	8.511104	-15.816969	-2.015189
H	8.053375	-14.105554	-2.198817
N	6.548925	-15.325216	-1.355758
H	5.707896	-15.766889	-1.702881
C	6.586662	-15.076611	-0.015360
O	5.553640	-15.388791	0.667668
O	7.644807	-14.541351	0.445559
N	10.007371	-15.572885	1.718153
C	10.688646	-15.284460	0.555389
C	10.605196	-16.620465	2.258696
H	10.281897	-17.123075	3.166916
N	11.651981	-17.011590	1.494186
H	12.262479	-17.798109	1.687149
C	11.723986	-16.173646	0.401135
H	12.477436	-16.286701	-0.373596
N	10.643825	-11.297514	3.073636
H	11.552414	-10.847693	3.091219
C	9.449485	-10.741189	3.479488
C	10.398753	-12.562483	2.663085
H	11.169802	-13.233781	2.293724
N	9.109697	-12.846820	2.779035
C	8.501239	-11.715245	3.291587
H	7.439362	-11.676594	3.531213
C	3.390566	-11.301200	5.186247
H	2.665805	-11.915293	5.742838
H	3.931756	-10.642463	5.877051
C	4.357566	-12.191988	4.418886
O	3.810922	-13.096699	3.682630
O	5.581183	-12.013826	4.537040
NI	8.190026	-14.707938	2.388808
NI	4.760451	-14.060761	2.125641
O	6.434281	-14.170512	3.143123
H	6.367746	-13.407844	3.760193
O	4.168010	-15.754549	4.953293
H	3.317645	-15.318350	5.100195
H	4.732904	-15.023408	4.643112
C	6.839239	-16.918605	3.790520
O	7.933836	-16.574869	3.270924
N	6.734204	-17.085994	5.131802
H	7.514758	-16.750372	5.682024
N	5.752513	-17.201023	3.039148
H	5.810402	-17.028344	5.555097
H	5.766952	-16.810877	2.094449
H	4.854588	-17.149917	3.517732
H	2.813120	-10.689753	4.473974
H	3.693863	-9.569726	-0.410597
H	-0.400435	-15.252425	1.801672
H	7.346345	-15.334164	-3.279553
H	10.371073	-14.478268	-0.100427
H	9.377608	-9.730377	3.871331

[1]PB

N	5.047222	-10.297472	0.213131
H	5.594134	-9.469050	0.008758
C	3.715614	-10.488462	-0.092365
C	5.500340	-11.404834	0.846197
H	6.510671	-11.558389	1.219249
N	4.528252	-12.293296	0.960772
C	3.408873	-11.739854	0.381573
H	2.458726	-12.266967	0.346303

N	1.542638	-16.272074	0.606788
H	1.132860	-16.966562	-0.008021
C	0.872662	-15.532873	1.559595
C	2.848747	-15.914375	0.636285
H	3.626318	-16.326595	-0.002812
N	3.049378	-14.987976	1.557985
C	1.827291	-14.737140	2.143811
H	1.734276	-14.003893	2.940133
C	7.714113	-14.718679	-2.484698
H	8.418127	-15.568590	-2.547759
H	8.286490	-13.830867	-2.177287
N	6.626970	-14.962023	-1.562292
H	5.724903	-15.248020	-1.918623
C	6.760150	-14.950266	-0.202477
O	5.722139	-15.260366	0.481503
O	7.897359	-14.640232	0.261747
N	10.000744	-15.780090	1.871969
C	10.710066	-15.892326	0.696085
C	10.490345	-16.683949	2.701435
H	10.121267	-16.875490	3.706447
N	11.496664	-17.369878	2.108281
H	12.027884	-18.123151	2.530999
C	11.651989	-16.883547	0.827172
H	12.390668	-17.281714	0.136906
N	11.138480	-11.345329	2.456836
H	12.057066	-10.944826	2.301751
C	10.077668	-10.731554	3.087968
C	10.743389	-12.585169	2.082773
H	11.389003	-13.292595	1.566812
N	9.486493	-12.796347	2.433652
C	9.054158	-11.646138	3.064760
H	8.049627	-11.553020	3.471954
C	3.549727	-12.157704	5.744364
H	3.216793	-13.051240	6.296171
H	4.046667	-11.460777	6.430845
C	4.487184	-12.564367	4.616267
O	4.017174	-13.477860	3.819477
O	5.601664	-12.041935	4.518246
NI	8.330378	-14.549531	2.229410
NI	4.872830	-14.046219	1.995812
O	6.669882	-13.577585	2.590723
H	6.530247	-13.033512	3.394392
O	3.503011	-15.842123	4.955507
H	2.584094	-15.986532	5.213232
H	3.551963	-14.922672	4.577669
C	6.609091	-16.248398	3.997768
O	7.778236	-16.027915	3.589487
N	6.307545	-16.218460	5.313956
H	7.017854	-15.858879	5.937763
N	5.608036	-16.568197	3.144796
H	5.325671	-16.195200	5.596017
H	5.787227	-16.483641	2.144564
H	4.654466	-16.638691	3.500741
H	2.649759	-11.679184	5.325486
H	3.118104	-9.738228	-0.602760
H	-0.195596	-15.633650	1.731220
H	7.304369	-14.538666	-3.488989
H	10.465335	-15.280727	-0.168560
H	10.141838	-9.726628	3.496103

^{2}R

N	5.584864	-10.633852	-0.383947
H	6.221958	-10.088132	-0.952495
C	4.277272	-10.308462	-0.087818
C	5.878553	-11.806129	0.229091
H	6.844720	-12.306435	0.180520
N	4.824571	-12.247606	0.893481
C	3.819529	-11.325090	0.712608
H	2.846438	-11.446373	1.180761
N	1.177290	-15.676273	0.730737
H	0.675620	-16.285972	0.094917
C	0.621366	-14.924836	1.746242
C	2.513869	-15.456321	0.727850
H	3.220201	-15.918519	0.043192
N	2.841037	-14.606494	1.686361
C	1.671822	-14.264245	2.333378
H	1.696102	-13.570464	3.169322
C	7.558307	-15.219023	-2.267144
H	8.362616	-15.937756	-2.032847
H	7.995619	-14.208059	-2.246518
N	6.441729	-15.326777	-1.352474
H	5.570794	-15.724415	-1.678121
C	6.520808	-15.068405	-0.014450
O	5.482757	-15.306560	0.690250
O	7.620533	-14.599314	0.418524
N	9.992772	-15.507701	1.786916

C	10.642471	-15.344944	0.582864
C	10.661335	-16.428707	2.457427
H	10.385564	-16.817104	3.434719
N	11.723808	-16.859647	1.736198
H	12.386834	-17.570052	2.026334
C	11.730827	-16.181742	0.535472
H	12.481613	-16.357285	-0.230149
N	10.498873	-11.071547	2.733275
H	11.369536	-10.560466	2.639999
C	9.326524	-10.594245	3.281495
C	10.293161	-12.354774	2.357764
H	11.058562	-12.976271	1.899204
N	9.050767	-12.723639	2.631255
C	8.430998	-11.631798	3.211244
H	7.402883	-11.669915	3.570868
C	3.514502	-11.307744	5.435680
H	2.819053	-11.974792	5.970020
H	4.093683	-10.725002	6.163156
C	4.436480	-12.135507	4.548459
O	3.848216	-12.819273	3.636931
O	5.663766	-12.111366	4.756577
NI	8.161594	-14.620242	2.372554
NI	4.750236	-13.874849	2.153390
O	6.434657	-14.055264	3.086935
H	6.362056	-13.414600	3.832753
C	6.806423	-16.994632	3.729499
O	7.868915	-16.455297	3.346674
N	6.735736	-17.531542	4.976404
H	7.506666	-17.351959	5.605636
N	5.735380	-17.134025	2.920451
H	5.875634	-17.891003	5.368165
H	5.699573	-16.556786	2.070294
H	4.852016	-17.459148	3.292588
H	2.901718	-10.633829	4.816579
H	3.801200	-9.404882	-0.458311
H	-0.444580	-14.927130	1.956919
H	7.204319	-15.418292	-3.288293
H	10.263740	-14.661980	-0.172781
H	9.233863	-9.586976	3.678084

[2]**TS1A** (-262i cm^{-1})

N	5.114959	-10.253204	0.162937
H	5.654685	-9.609375	-0.403676
C	3.908550	-9.983510	0.774596
C	5.460660	-11.531668	0.454285
H	6.374792	-12.017985	0.114248
N	4.537180	-12.091156	1.216358
C	3.564543	-11.140548	1.428278
H	2.692500	-11.349949	2.041861
N	0.874804	-15.265203	0.299140
H	0.339887	-15.670182	-0.460663
C	0.366570	-14.805816	1.496713
C	2.217111	-15.078350	0.312599
H	2.892497	-15.349528	-0.494772
N	2.591371	-14.525635	1.451947
C	1.450558	-14.348587	2.204628
H	1.507129	-13.905364	3.195081
C	7.313048	-14.842538	-2.738601
H	7.788511	-13.852059	-2.674348
H	6.951291	-14.985321	-3.766785
N	6.191954	-14.935678	-1.828554
H	5.337747	-15.377012	-2.142224
C	6.262264	-14.652338	-0.492069
O	5.238469	-14.894167	0.211788
O	7.361629	-14.141958	-0.079792
N	9.725247	-15.547545	1.042400
C	10.506555	-15.389843	-0.081183
C	10.085284	-16.690312	1.603165
H	9.621133	-17.119623	2.489106
N	11.078135	-17.268145	0.888008
H	11.522353	-18.153719	1.105234
C	11.363278	-16.457968	-0.190535
H	12.122465	-16.709382	-0.926157
N	10.648189	-11.091205	2.290138
H	11.540602	-10.617563	2.201271
C	9.536756	-10.615902	2.952613
C	10.351290	-12.314897	1.794838
H	11.056378	-12.924903	1.234392
N	9.107153	-12.647214	2.099911
C	8.583868	-11.595116	2.826305
H	7.575497	-11.617745	3.233142
C	3.525599	-11.907368	5.722052
H	2.827495	-12.616176	6.194260
H	4.153254	-11.435239	6.488215
C	4.375796	-12.629764	4.689313
O	3.750267	-13.261643	3.780022

O	5.629076	-12.573366	4.795868
NI	8.109589	-14.449432	1.748061
NI	4.546281	-14.033381	2.017955
O	6.459059	-13.955760	2.826284
H	6.285563	-13.349004	3.628484
C	6.426013	-15.632810	3.379012
O	7.441980	-16.094342	2.732682
N	6.477565	-15.632654	4.771730
H	7.424353	-15.478569	5.107218
N	5.100203	-15.963576	2.878040
H	5.833677	-14.983949	5.221521
H	5.203721	-16.571649	2.065487
H	4.534538	-16.425510	3.589351
H	2.913502	-11.140222	5.221709
H	3.414515	-9.019162	0.694619
H	-0.692636	-14.845159	1.736046
H	8.090333	-15.601442	-2.536925
H	10.382052	-14.540472	-0.748441
H	9.520863	-9.652569	3.454650

^{2}Int1A

N	5.024387	-10.236499	0.079685
H	5.541678	-9.602492	-0.518188
C	3.816896	-9.973491	0.691868
C	5.405614	-11.493138	0.417071
H	6.327460	-11.971287	0.085446
N	4.504247	-12.045340	1.210958
C	3.508529	-11.111608	1.394234
H	2.646423	-11.317793	2.022846
N	0.865940	-15.195454	0.272081
H	0.338042	-15.586537	-0.499815
C	0.349963	-14.781163	1.482871
C	2.205873	-14.994989	0.294997
H	2.887536	-15.234939	-0.517013
N	2.570989	-14.475568	1.452794
C	1.427161	-14.334620	2.207800
H	1.472953	-13.920920	3.211522
C	7.392622	-14.897564	-2.666691
H	7.819182	-13.882434	-2.673497
H	7.067423	-15.141812	-3.687910
N	6.252274	-14.976613	-1.779641
H	5.409047	-15.435222	-2.097923
C	6.267527	-14.589262	-0.468169
O	5.218129	-14.792387	0.209505
O	7.340151	-14.025417	-0.054216
N	9.728495	-15.508035	1.008867
C	10.526129	-15.372217	-0.106244
C	10.034027	-16.670589	1.561090
H	9.538899	-17.086142	2.437276
N	11.007674	-17.282813	0.848647
H	11.412690	-18.188038	1.061324
C	11.337286	-16.474980	-0.219215
H	12.093611	-16.750623	-0.949103
N	10.613210	-10.956676	2.132116
H	11.485689	-10.458065	1.994857
C	9.515583	-10.499638	2.829095
C	10.336020	-12.201665	1.680099
H	11.039274	-12.805621	1.110833
N	9.117800	-12.565809	2.046270
C	8.591105	-11.511974	2.767245
H	7.602158	-11.553554	3.217968
C	3.548557	-11.813185	5.718931
H	2.825867	-11.142943	5.228969
H	2.962430	-12.545304	6.297783
C	4.366547	-12.542012	4.670294
O	3.732994	-13.185598	3.783096
O	5.628700	-12.486634	4.751588
NI	8.149190	-14.385897	1.740327
NI	4.519019	-13.988556	2.021922
O	6.475458	-13.934578	2.933529
H	6.219227	-13.284236	3.731431
C	6.456343	-15.402663	3.383809
O	7.457350	-15.957448	2.720965
N	6.547086	-15.525845	4.800096
H	7.520176	-15.458743	5.088975
N	5.108364	-15.828506	2.920319
H	6.002285	-14.825856	5.302986
H	5.244138	-16.526966	2.188224
H	4.589600	-16.261199	3.684760
H	4.194184	-11.242735	6.398215
H	3.297818	-9.025777	0.578452
H	-0.708819	-14.840710	1.719636
H	8.198418	-15.597497	-2.383531
H	10.448380	-14.510118	-0.764113
H	9.488135	-9.524383	3.307039

^{2}TS2A (-304*i* cm^{-1})

N	5.195687	-10.234915	0.247375
H	5.760988	-9.591008	-0.293813
C	3.941037	-9.982499	0.761524
C	5.544768	-11.501387	0.581763
H	6.479865	-11.984936	0.297185
N	4.578906	-12.070778	1.282533
C	3.572617	-11.138565	1.403939
H	2.652640	-11.359989	1.938474
N	0.950031	-15.166278	0.179017
H	0.444683	-15.526156	-0.622619
C	0.403076	-14.818057	1.395955
C	2.286019	-14.948346	0.239964
H	2.989292	-15.139289	-0.566275
N	2.622521	-14.478718	1.427783
C	1.459583	-14.391655	2.162578
H	1.467920	-14.027443	3.185684
C	7.317374	-14.797524	-2.780617
H	7.906269	-13.892619	-2.578223
H	6.952379	-14.750614	-3.817549
N	6.190969	-14.857697	-1.875044
H	5.362942	-15.362852	-2.163672
C	6.259973	-14.553467	-0.539713
O	5.231605	-14.797703	0.159129
O	7.344511	-14.022762	-0.124831
N	9.673446	-15.585768	0.933837
C	10.474762	-15.460618	-0.178253
C	9.989925	-16.733886	1.507472
H	9.493429	-17.133844	2.390445
N	10.974581	-17.349129	0.809927
H	11.388437	-18.246051	1.038841
C	11.299930	-16.555511	-0.269886
H	12.062266	-16.834531	-0.992174
N	10.583151	-11.070339	2.234031
H	11.442171	-10.548095	2.101326
C	9.522180	-10.703124	3.034768
C	10.287873	-12.265716	1.668215
H	10.959550	-12.795834	0.996239
N	9.096052	-12.679539	2.060733
C	8.603307	-11.716742	2.916559
H	7.632971	-11.841034	3.390922
C	3.333784	-11.548256	5.549919
H	3.298943	-11.834989	6.612314
H	3.831469	-10.567457	5.499010
C	4.139739	-12.560617	4.780883
O	3.672980	-13.214336	3.849456
O	5.374165	-12.675094	5.206681
NI	8.080031	-14.471530	1.700497
NI	4.609637	-14.045654	2.006984
O	6.471901	-14.008127	2.913867
H	5.829092	-13.428109	4.737905
C	6.459526	-15.350444	3.355470
O	7.447192	-16.022651	2.737420
N	6.526803	-15.326106	4.822135
H	7.481495	-15.052088	5.065836
N	5.102817	-15.875862	2.973686
H	6.421966	-16.273661	5.192194
H	5.224721	-16.680578	2.359268
H	4.534478	-16.140503	3.777979
H	2.317367	-11.475326	5.146649
H	3.433406	-9.031329	0.626959
H	-0.659765	-14.902446	1.605331
H	7.993606	-15.668355	-2.690908
H	10.389275	-14.611666	-0.852454
H	9.518111	-9.781879	3.610893

^{2}Int2A

N	5.156467	-10.182923	0.217720
H	5.726338	-9.504604	-0.274302
C	3.844728	-10.013639	0.609077
C	5.555328	-11.421232	0.598839
H	6.544151	-11.837325	0.408518
N	4.567675	-12.050909	1.211243
C	3.495647	-11.188452	1.227863
H	2.548245	-11.465840	1.681708
N	1.203941	-15.657723	0.294592
H	0.769343	-16.211895	-0.434517
C	0.564392	-15.095965	1.379186
C	2.520172	-15.344540	0.372936
H	3.282958	-15.655325	-0.336656
N	2.756543	-14.613332	1.447519
C	1.547595	-14.450203	2.087985
H	1.474955	-13.886392	3.013249
C	7.102652	-14.112464	-2.901167
H	7.829065	-13.387133	-2.512253
H	6.629517	-13.687260	-3.799871
N	6.101807	-14.369825	-1.889823

H	5.214299	-14.764312	-2.173678
C	6.339743	-14.368147	-0.538729
O	5.403908	-14.777363	0.213819
O	7.476924	-13.937260	-0.153262
N	9.600044	-15.749280	0.910905
C	10.286737	-15.778795	-0.281710
C	9.926821	-16.846524	1.571694
H	9.516160	-17.124453	2.541358
N	10.808211	-17.579648	0.850520
H	11.208208	-18.467669	1.132655
C	11.052652	-16.918164	-0.335040
H	11.724566	-17.308946	-1.094320
N	10.933136	-11.278187	2.062061
H	11.827522	-10.830191	1.895976
C	9.903769	-10.780121	2.832899
C	10.548171	-12.482365	1.573635
H	11.173156	-13.103390	0.934998
N	9.329940	-12.777361	1.989540
C	8.910640	-11.727216	2.778009
H	7.928744	-11.751214	3.245593
C	2.856311	-11.745667	5.553182
H	2.262382	-12.253025	6.330522
H	3.404647	-10.932348	6.050690
C	3.829873	-12.731709	4.952199
O	3.691194	-13.149336	3.798480
O	4.771454	-13.094218	5.770481
NI	8.161856	-14.456959	1.680251
NI	4.687946	-13.975053	2.033052
O	6.575953	-13.807815	2.837032
H	5.448779	-13.804546	5.391337
C	6.592086	-15.039236	3.486741
O	7.636740	-15.768819	3.068529
N	6.583803	-14.788670	4.956060
H	7.445507	-14.272875	5.153065
N	5.279579	-15.698211	3.146184
H	6.685397	-15.681269	5.448116
H	5.482401	-16.533191	2.596645
H	4.724242	-15.963484	3.959243
H	2.186071	-11.341036	4.785592
H	3.290446	-9.098755	0.418813
H	-0.503692	-15.202165	1.548082
H	7.656725	-15.021574	-3.203408
H	10.165731	-14.996338	-1.027019
H	9.969688	-9.824160	3.345214

^{2}TS3A (-87*i* cm^{-1})

N	5.255015	-10.179494	0.095292
H	5.785121	-9.571037	-0.517435
C	4.067435	-9.867785	0.724627
C	5.593037	-11.447959	0.435127
H	6.493235	-11.957874	0.092665
N	4.683451	-11.960979	1.245281
C	3.728490	-10.989615	1.439600
H	2.875168	-11.160931	2.089146
N	1.117275	-15.341505	0.309013
H	0.646050	-15.854671	-0.427150
C	0.515923	-14.738894	1.393851
C	2.453099	-15.130444	0.398091
H	3.192640	-15.492228	-0.311059
N	2.738105	-14.427030	1.479140
C	1.540947	-14.175028	2.112698
H	1.507709	-13.608803	3.038396
C	7.184041	-14.602026	-2.844753
H	7.842633	-13.750920	-2.623611
H	6.733876	-14.445028	-3.836324
N	6.139258	-14.685721	-1.847928
H	5.248890	-15.088486	-2.109525
C	6.336050	-14.505156	-0.502527
O	5.367718	-14.764672	0.269012
O	7.483472	-14.063796	-0.150407
N	9.717537	-15.688777	0.959611
C	10.428964	-15.682530	-0.218977
C	10.057044	-16.789321	1.608733
H	9.633494	-17.098362	2.562939
N	10.970668	-17.488734	0.894788
H	11.385402	-18.372204	1.170301
C	11.223154	-16.802126	-0.274313
H	11.919633	-17.164026	-1.025725
N	10.821626	-11.107370	1.905080
H	11.696822	-10.626360	1.728792
C	9.747021	-10.609512	2.611602
C	10.512021	-12.361426	1.496695
H	11.185966	-12.992595	0.920939
N	9.298142	-12.689303	1.902614
C	8.804517	-11.608183	2.601897
H	7.812710	-11.645215	3.046923

C	2.755468	-12.215116	5.788936
H	2.048500	-12.894901	6.291910
H	3.246734	-11.628247	6.579006
C	3.790012	-13.041743	5.043332
O	3.766246	-13.038119	3.788616
O	4.591427	-13.702223	5.775101
NI	8.218137	-14.444541	1.681245
NI	4.693149	-13.876516	2.090679
O	6.633274	-13.827996	2.852673
H	5.663704	-14.306883	5.320719
C	6.612795	-15.074858	3.431870
O	7.631576	-15.822469	3.019357
N	6.650310	-14.863285	4.949326
H	7.460898	-14.269024	5.139910
N	5.270433	-15.675322	3.138257
H	6.805674	-15.755555	5.428031
H	5.411207	-16.494696	2.547764
H	4.728992	-15.940970	3.960732
H	2.202953	-11.551237	5.111836
H	3.582502	-8.901909	0.613550
H	-0.558286	-14.762912	1.555258
H	7.811751	-15.511907	-2.892340
H	10.302714	-14.892974	-0.955751
H	9.749575	-9.616864	3.053547

^{2}Int3A

N	5.233592	-10.175140	0.086250
H	5.755098	-9.565150	-0.532288
C	4.052158	-9.866791	0.728892
C	5.578480	-11.442942	0.421769
H	6.476860	-11.949935	0.070276
N	4.679627	-11.958071	1.242438
C	3.724586	-10.989176	1.448270
H	2.880777	-11.163517	2.109312
N	1.117732	-15.293595	0.279872
H	0.643162	-15.776829	-0.474160
C	0.522041	-14.740623	1.393898
C	2.453360	-15.081300	0.368686
H	3.189001	-15.410985	-0.359651
N	2.743568	-14.423275	1.476512
C	1.550017	-14.203176	2.128521
H	1.520449	-13.673285	3.075527
C	7.202423	-14.634564	-2.837703
H	7.849864	-13.770867	-2.633010
H	6.755767	-14.505541	-3.834851
N	6.152919	-14.707574	-1.844869
H	5.270032	-15.129118	-2.102323
C	6.343184	-14.504280	-0.501647
O	5.374070	-14.757642	0.270302
O	7.486910	-14.050416	-0.152304
N	9.710567	-15.690182	0.951228
C	10.425595	-15.680187	-0.225170
C	10.046331	-16.794067	1.596746
H	9.620265	-17.106196	2.548792
N	10.961120	-17.491804	0.882745
H	11.373660	-18.377081	1.155818
C	11.218228	-16.800763	-0.282673
H	11.916462	-17.160570	-1.033462
N	10.833613	-11.111171	1.895906
H	11.707949	-10.630901	1.713369
C	9.765714	-10.613515	2.612558
C	10.519017	-12.363826	1.487242
H	11.187584	-12.994612	0.904886
N	9.307739	-12.690700	1.901792
C	8.821045	-11.610124	2.606907
H	7.831446	-11.645173	3.056957
C	2.745361	-12.248913	5.793884
H	2.038324	-12.926761	6.299069
H	3.244748	-11.668452	6.583976
C	3.774150	-13.077571	5.040423
O	3.769575	-13.030396	3.784116
O	4.547943	-13.778473	5.759961
NI	8.219631	-14.439157	1.677586
NI	4.697979	-13.871666	2.090803
O	6.639655	-13.816723	2.859550
H	5.706399	-14.339186	5.325805
C	6.620182	-15.062368	3.436755
O	7.629679	-15.815878	3.018582
N	6.683583	-14.844585	4.960792
H	7.479168	-14.226345	5.137075
N	5.272150	-15.656317	3.168745
H	6.863370	-15.731832	5.440065
H	5.397359	-16.507417	2.621621
H	4.719661	-15.860026	4.001646
H	2.193388	-11.576944	5.124222
H	3.563412	-8.902227	0.623227

H	-0.550809	-14.776184	1.562018
H	7.840892	-15.537866	-2.861052
H	10.302715	-14.887517	-0.959162
H	9.773215	-9.621851	3.056644

^{2}TS4A (-216*i* cm^{-1})

N	5.020723	-10.152959	-0.016178
H	5.537654	-9.450890	-0.532545
C	3.695317	-10.077318	0.359729
C	5.519064	-11.331025	0.431815
H	6.545192	-11.659253	0.273714
N	4.582542	-12.012579	1.069383
C	3.441636	-11.242614	1.038277
H	2.527645	-11.566253	1.526262
N	1.671434	-16.202864	0.473430
H	1.362762	-16.947889	-0.140748
C	0.880064	-15.494874	1.353555
C	2.940053	-15.737872	0.579606
H	3.793685	-16.113753	0.020066
N	2.999615	-14.771078	1.478514
C	1.725228	-14.608156	1.974032
H	1.517478	-13.871981	2.745345
C	6.727784	-13.583914	-2.826019
H	7.548860	-12.952317	-2.464086
H	6.072597	-12.977152	-3.471055
N	5.991362	-14.089109	-1.687754
H	5.077519	-14.497845	-1.836790
C	6.481899	-14.201179	-0.418817
O	5.768453	-14.782351	0.456734
O	7.636694	-13.705654	-0.176212
N	9.443042	-15.808731	0.875937
C	10.038373	-15.915257	-0.361635
C	9.712084	-16.924577	1.533655
H	9.357089	-17.152668	2.537274
N	10.467219	-17.744323	0.766073
H	10.801638	-18.663167	1.035508
C	10.688182	-17.122214	-0.445417
H	11.266372	-17.583114	-1.241611
N	11.398454	-11.648843	2.245087
H	12.363114	-11.353798	2.140354
C	10.398964	-10.948424	2.885810
C	10.871474	-12.806991	1.780963
H	11.440422	-13.554637	1.232052
N	9.589617	-12.882303	2.091971
C	9.275361	-11.731019	2.781865
H	8.264752	-11.562398	3.146528
C	2.688801	-11.927668	5.553889
H	1.778082	-12.473200	5.853338
H	3.156171	-11.553378	6.475822
C	3.626609	-12.897032	4.847488
O	3.628591	-12.869421	3.577855
O	4.303346	-13.682566	5.558158
NI	8.218371	-14.340476	1.654746
NI	4.753058	-13.824697	2.124719
O	6.699410	-13.545070	2.910670
H	7.317518	-14.944285	5.920598
C	6.749573	-14.714881	3.555663
O	7.749000	-15.478719	3.276043
N	7.013251	-14.166858	5.332217
H	6.105494	-13.792009	5.668303
N	5.444236	-15.316503	3.628756
H	7.733219	-13.444485	5.331716
H	5.500847	-16.333005	3.625879
H	4.883201	-14.944853	4.420174
H	2.400993	-11.093478	4.900475
H	3.068271	-9.221895	0.124412
H	-0.185332	-15.679556	1.460199
H	7.162630	-14.391580	-3.443612
H	9.939456	-15.128660	-1.105627
H	10.569821	-9.980386	3.348799

^{2}PA

N	5.567591	-10.369428	-0.636614
H	5.923250	-9.992984	-1.507678
C	4.854385	-9.675487	0.320082
C	5.706067	-11.649989	-0.217169
H	6.263348	-12.421340	-0.747658
N	5.117165	-11.808172	0.956163
C	4.582552	-10.587332	1.308571
H	4.063117	-10.465265	2.254953
N	0.895184	-14.471495	0.698001
H	0.264111	-15.006622	0.112348
C	0.532310	-13.509512	1.618141
C	2.243241	-14.589287	0.719287
H	2.821090	-15.270543	0.100841
N	2.761431	-13.754852	1.604375

C	1.708103	-13.074722	2.175464
H	1.874553	-12.324572	2.942764
C	6.985176	-16.476328	-2.113279
H	7.696591	-15.652460	-2.248548
H	6.544970	-16.725660	-3.091648
N	5.953326	-16.055500	-1.190854
H	5.112373	-16.614400	-1.112968
C	6.105774	-15.099510	-0.227082
O	5.139525	-14.935504	0.576810
O	7.191611	-14.419777	-0.235097
N	10.043466	-14.961640	0.617618
C	10.720009	-14.764784	-0.566210
C	10.649863	-15.956465	1.246589
H	10.330065	-16.375066	2.199793
N	11.697829	-16.398243	0.514413
H	12.319972	-17.156100	0.774676
C	11.762176	-15.655577	-0.645036
H	12.517517	-15.824894	-1.407523
N	9.998075	-10.280669	1.606146
H	10.730118	-9.601542	1.428232
C	8.899647	-10.098850	2.421104
C	9.947716	-11.535628	1.104627
H	10.704817	-11.951391	0.443028
N	8.871594	-12.164646	1.550526
C	8.206158	-11.281860	2.378831
H	7.288593	-11.568340	2.887505
C	3.577188	-12.046022	5.891665
H	2.483641	-11.925516	5.819647
H	3.807716	-12.475226	6.876045
C	4.055239	-12.971061	4.782431
O	4.307528	-12.412408	3.655044
O	4.153984	-14.190971	5.002037
NI	8.311700	-14.156210	1.400028
NI	4.791384	-13.533907	2.044177
O	6.713710	-13.979691	2.715616
H	4.761952	-18.319879	5.936971
C	7.020290	-15.167195	3.204343
O	8.077372	-15.703363	2.734045
N	5.348790	-17.505960	6.131469
H	4.704060	-16.723324	6.286353
N	6.231117	-15.721416	4.103344
H	5.786947	-17.689949	7.036436
H	6.406122	-16.612229	4.577425

H	5.436101	-15.161896	4.460903
H	4.033432	-11.050588	5.794589
H	4.607320	-8.622605	0.215611
H	-0.502443	-13.228571	1.793246
H	7.543892	-17.358416	-1.750372
H	10.404712	-14.018976	-1.292258
H	8.715623	-9.170424	2.954481

Section 8: Key bond distance for all stationary points obtained from QM-only cluster and QM/MM metadynamics calculations.

Table S1. Key bond distances [in Å] for ^{1}**R**, ^{1}**TS1A**, and ^{1}**Int1A**.

Parameters	^{1}R			^{1}TS1A			^{1}Int1A		
	QM1	QM2	QM1/MM MD	QM1	QM2	QM1/MM MD	QM1	QM2	QM1/MM MD
Ni1-O(WB)	2.02	1.96	1.98	2.14	2.14	2.19	2.24	2.01	2.42
Ni2-O(WB)	2.13	2.04	2.12	2.13	2.23	2.12	2.21	2.04	2.05
Ni1-O(urea)	2.11	2.10	2.33	2.04	1.95	1.97	2.03	2.01	2.06
Ni2-O(W2)	2.13	2.28	2.24	2.27	2.10	1.98	2.24	2.11	2.11
O(WB)-C(urea)	3.30	3.39	3.15	1.93	1.55	1.98	1.49	1.39	1.45
C(urea)-O(urea)	1.24	1.25	1.22	1.27	1.33	1.27	1.33	1.35	1.30
C(urea)-N1(urea)	1.36	1.36	1.32	1.41	1.40	1.44	1.46	1.50	1.49
C(urea)-N2(urea)	1.34	1.35	1.40	1.37	1.48	1.42	1.43	1.50	1.47

QM1 = GFN2-xTB, QM2 = UB3LYP/def2-SVP.

Table S2. Key bond distances [in Å] for ^{2}**R**, ^{2}**TS1A**, and ^{2}**Int1A**.

Parameters	^{2}R			^{2}TS1A			^{2}Int1A		
	QM1	QM2	QM1/MM MD	QM1	QM2	QM1/MM MD	QM1	QM2	QM1/MM MD
Ni1-O(WB)	2.01	1.95	2.01	2.11	2.03	2.12	2.17	2.10	2.28
Ni2-O(WB)	2.08	1.93	2.19	2.13	2.08	2.00	2.25	2.16	2.32
Ni1-O(urea)	2.13	2.10	2.25	2.02	2.03	2.12	2.01	1.98	1.96
Ni2-N2(urea)	2.33	3.49	2.92	2.69	2.18	2.58	2.18	2.13	2.16
O(WB)-C(urea)	2.59	3.03	2.73	1.80	1.77	1.96	1.48	1.54	1.35
C(urea)-O(urea)	1.23	1.25	1.29	1.28	1.29	1.35	1.32	1.32	1.32
C(urea)-N1(urea)	1.35	1.36	1.35	1.39	1.39	1.34	1.43	1.42	1.41
C(urea)-N2(urea)	1.38	1.35	1.34	1.40	1.46	1.35	1.49	1.49	1.50

QM1 = GFN2-xTB, QM2 = UB3LYP/def2-SVP.

Table S3. Key bond distances [in Å] for **^{1}TS2A**, **^{2}TS2A**, **^{2}Int2A** and **^{2}TS3A**.

Parameters	**^{1}TS2A**			**^{2}TS2A**			**^{2}Int2A**	**^{2}TS3A**
	QM1	QM2	QM1/MM MD	QM1	QM2	QM1/MM MD	QM2	QM2
Ni1-O(WB)	2.09	2.01	2.07	2.11	2.07	1.98	2.07	2.06
Ni2-O(WB)	2.10	2.05	2.14	2.08	2.07	2.12	2.06	2.09
Ni1-O(urea)	2.03	2.02	2.03	2.01	1.97	2.09	1.98	2.01
Ni2-O(W2)<br>(Ni2-N2(urea))	2.21	2.11	2.25	(2.64)	(2.13)	(2.14)	(2.14)	(2.16)
O(WB)-C(urea)	1.34	1.38	1.38	1.37	1.41	1.35	1.39	1.37
C(urea)-O(urea)	1.33	1.34	1.34	1.33	1.35	1.34	1.34	1.33
C(urea)-N1(urea)	1.55	1.52	1.46	1.54	1.47	1.46	1.49	1.53
C(urea)-N2(urea)	1.48	1.50	1.47	1.46	1.50	1.35	1.51	1.50
O(WB)-H(WB)	2.37	2.49	1.99	2.40	2.02	2.11	2.79	2.69
H(WB)-O(D363)	1.29	1.19	0.99	1.24	1.00	0.96	1.05	1.31
H(WB)-N1(urea)	1.25	1.31	1.82	1.30	2.02	1.77	1.56	1.19

QM1 = GFN2-xTB, QM2 = UB3LYP/def2-SVP.

Table S4. Key bond distances [in Å] for **^{2}int3A** and **^{2}TS4A**.

Parameters	**^{2}Int3A**		**^{2}TS4A**	
	QM1	QM2	QM1	QM2
Ni1-O(WB)	2.11	2.07	2.11	2.13
Ni2-O(WB)	2.09	2.09	2.09	2.12
Ni1-O(urea)	2.03	2.01	2.04	2.04
Ni2-N2(urea)	2.73	2.16	2.93	2.23
O(WB)-C(urea)	1.34	1.37	1.31	1.34
C(urea)-O(urea)	1.31	1.33	1.29	1.29
C(urea)-N1(urea)	1.63	1.54	1.87	1.88
C(urea)-N2(urea)	1.45	1.50	1.41	1.44
H(WB)-O(D363)	1.48	1.36	1.71	1.81
H(WB)-N1(urea)	1.11	1.16	1.04	1.04

QM1 = GFN2-xTB, QM2 = UB3LYP/def2-SVP.

Table S5. Key bond distances [in Å] for **[1]PA** and **[2]PA**.

Parameters	^{1}**PA**			^{2}**PA**		
	QM1	QM2	QM1/MM MD	QM1	QM2	QM1/MM MD
Ni1-O(WB)	2.18	2.10	2.25	2.17	2.10	1.97
Ni2-O(WB)	2.15	2.17	2.25	2.14	2.10	2.16
Ni1-O(urea)	2.08	2.08	1.91	2.07	2.07	1.99
Ni2-O(W2) (Ni2-N2(urea))	2.25	2.15	2.55	(3.77)	(3.78)	(2.22)
O(WB)-C(urea)	1.27	1.29	1.26	1.28	1.30	1.23
C(urea)-O(urea)	1.26	1.27	1.33	1.27	1.27	1.27
C(urea)-N2(urea)	1.35	1.37	1.37	1.33	1.34	1.43

QM1 = GFN2-xTB, QM2 = UB3LYP/def2-SVP.

Section 9: Full citation of ref. 59

(59) Frisch, M. J.; Trucks, G. W.; Schlegel, H. B.; Scuseria, G. E.; Robb, M. A.; Cheeseman, J. R.; Scalmani, G.; Barone, V.; Petersson, G. A.; Nakatsuji, H.; Li, X.; Caricato, M.; Marenich, A. V.; Bloino, J.; Janesko, B. G.; Gomperts, R.; Mennucci, B.; Hratchian, H. P.; Ortiz, J. V.; Izmaylov, A. F.; Sonnenberg, J. L.; Williams-Young, D.; Ding, F.; Lipparini, F.; Egidi, F.; Goings, J.; Peng, B.; Petrone, A.; Henderson, T.; Ranasinghe, D.; Zakrzewski, V. G.; Gao, J.; Rega, N.; Zheng, G.; Liang, W.; Hada, M.; Ehara, M.; Toyota, K.; Fukuda, R.; Hasegawa, J.; Ishida, M.; Nakajima, T.; Honda, Y.; Kitao, O.; Nakai, H.; Vreven, T.; Throssell, K.; Montgomery, J. A., Jr.; Peralta, J. E.; Ogliaro, F.; Bearpark, M. J.; Heyd, J. J.; Brothers, E. N.; Kudin, K. N.; Staroverov, V. N.; Keith, T. A.; Kobayashi, R.; Normand, J.; Raghavachari, K.; Rendell, A. P.; Burant, J. C.; Iyengar, S. S.; Tomasi, J.; Cossi, M.; Millam, J. M.; Klene, M.; Adamo, C.; Cammi, R.; Ochterski, J. W.; Martin, R. L.; Morokuma, K.; Farkas, O.; Foresman, J. B.; Fox, D. J. *Gaussian 16*, Revision B.01; Gaussian, Inc., Wallingford CT, 2016.
